# Supplementary material for: Role of Açaí (Euterpe oleracea) in Modulating the Immune Response During Experimental Oral Infection with Trypanosoma cruzi
Source: Microorganisms. 2025 Nov 28;13(12):2711. doi: 10.3390/microorganisms13122711 (PMC12735136; doi:10.3390/microorganisms13122711)
Supplement: Supplementary file 1 [file microorganisms-13-02711-s001.zip › microorganisms-3981490-Supplementary File S1.pdf]

Supplementary Material File S1- Characterization of açaí pulp

The centesimal composition of açaí pulp was determined in collaboration with the Multidisciplinary Food Analysis Laboratory at the School of Nutrition (ENUT), Federal University of Ouro Preto (UFOP). Flowchart S1 summarizes the analyses conducted and the methodologies applied.

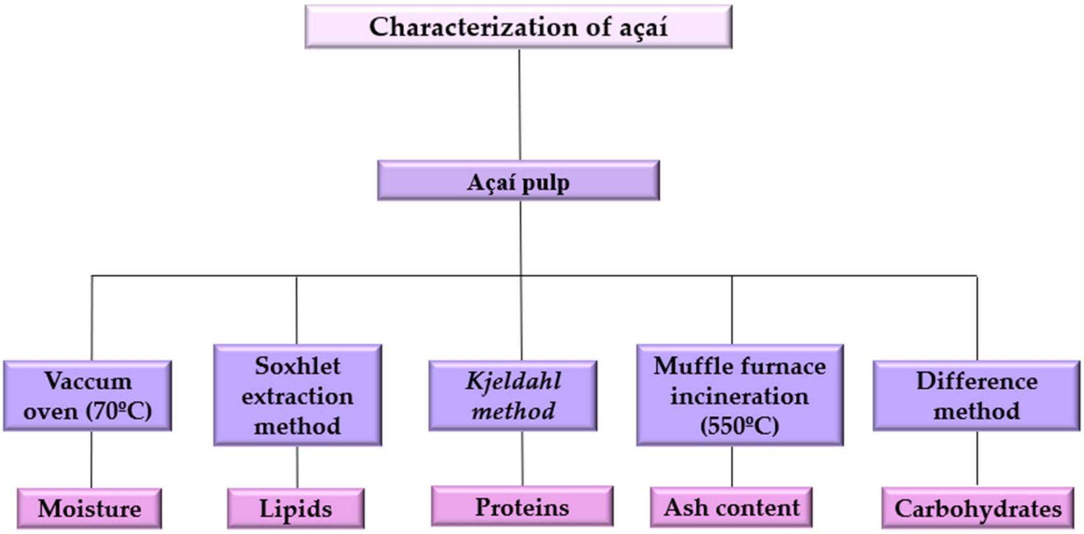

Flowchart S1. Characterization of Açaí

The results of the characterization of açaí pulp are presented in Table S4. As expected, the major component of açaí pulp was moisture, at 88.33% (±1.15), resulting in a total solids (dry matter) content of 11.67%. The centesimal composition of macronutrients in the dry matter was 51.45% carbohydrates, 39.13% lipids, and 5.26% protein. The ash content was 4.16%.

Table S4. Nutritional composition of açaí

**Composition**	**100 g of pulp**	**100 g of dry matter**
pH	4,8	-
Moisture	88,33	-
Carbohydrates	4,26	51,45
Lipids	4,57	39,13
Proteins	1,96	5,26
Ash content	0,48	4,16

Values are presented as mean ± standard deviation.

Total polyphenols were quantified in the hydroalcoholic extract of açaí using the Folin–Ciocalteu method. In this assay, phenolic compounds react with Folin–Ciocalteu reagent, forming a blue complex that is measured by spectrophotometry. Quantification was performed using a gallic acid standard curve, and results were expressed as gallic acid equivalents (GAE). Antioxidant activity of the hydroalcoholic extract of açaí was quantified using two methodologies: the DPPH• radical scavenging assay and the ABTS•+ radical cation assay. In the DPPH• assay, antioxidant activity is measured by the ability of compounds in the extract to donate electrons or hydrogen atoms to neutralize the purple DPPH• radical. The decrease in absorbance is inversely proportional to the antioxidant content. The ABTS assay measures antioxidant capacity by assessing the ability of compounds to reduce the ABTS•+ radical cation, generated by the oxidation of ABTS. Antioxidants quench the blue-green ABTS•+ radical, causing a decrease in absorbance. Results are expressed as Trolox equivalents, using a calibration curve constructed with the synthetic antioxidant Trolox.

The results for total polyphenol quantification and antioxidant capacity of açaí are summarized in Table S5. The polyphenol content was 198.77 ± 1.63 mg GAE per 100 g açaí.

The antioxidant capacity measured by the DPPH method was evaluated using two parameters: EC50 and IAA. The EC50 value, which indicates the amount of açaí (in grams) needed to lower the initial DPPH• concentration by 50%, was 46,360.33 ± 76.57 grams of fruit per gram of DPPH•. The calculated Antioxidant Activity Index (IAA) was 0.00018.

On the other hand, the antioxidant capacity measured by the ABTS method, which indicates the ability of 1 g of açaí to inhibit the ABTS+• radical compared to the Trolox standard, was 6.74 ± 0.27.

Table S5. Polyphenols and Antioxidant Capacity of Açaí Hydroalcoholic Extract

**Total polyphenols (mg GAE /100 g de açaí)**	**DPPH (g of fruit/g de DPPH)**	**AAI**	**ABTS (μmol Trolox/g)**
198,77 ± 1,63	46360,33 ± 76,57	0,00018 ± 0,000005	6,74 ± 0,27

Results expressed as mean ± standard deviation. AAI – Antioxidant Activity Index.
