# Supplementary material for: Role of Açaí (Euterpe oleracea) in Modulating the Immune Response During Experimental Oral Infection with Trypanosoma cruzi
Source: Microorganisms. 2025 Nov 28;13(12):2711. doi: 10.3390/microorganisms13122711 (PMC12735136; doi:10.3390/microorganisms13122711)
Supplement: Supplementary file 1 [file microorganisms-13-02711-s001.zip › Table S1.pdf]

Table S1. Compositional analyis of stomach proteome during *Trypanosoma cruzi* infection.

Accession	Description	Coverage (%)	Unique Peptides	Molecular Mass (Da)	Control Area	RPMI Area	Açaí Area
Q64433	*10 kDa heat shock protein mitochondrial*	68	8	10963	1,98E+07	2,62E+07	2,66E+07
Q9CQV8	*14-3-3 protein beta/alpha*	39	4	28086	1,68E+07	1,58E+07	9,50E+06
P62259	*14-3-3 protein epsilon*	37	7	29,174	2,86E+07	2,25E+07	2,52E+07
P68510	*14-3-3 protein eta*	37	4	28,212	8,53E+06	8,24E+06	1,21E+08
P61982	*14-3-3 protein gamma*	46	6	28,303	9,48E+06	7,30E+06	6,13E+06
O70456	*14-3-3 protein sigma*	40	6	27,706	8,22E+06	8,43E+05	8,82E+06
P68254	*14-3-3 protein theta*	39	4	27,778	1,29E+07	8,02E+06	6,89E+06
P63101	*14-3-3 protein zeta/delta*	53	8	27,771	6,87E+07	5,68E+07	5,59E+07
A8Y5N4	*17-beta-hydroxysteroid dehydrogenase 13*	26	4	29425	4,37E+06		1,29E+06
Q99N15	*17beta-hydroxysteroid dehydrogenase type 10/short chain*	27	4	27,274	7,57E+05	4,61E+05	4,62E+05
Q9CQ62	*2 4-dienoyl-CoA reductase [(3E)-enoyl-CoA-producing] mitochondrial*	15	5	36214	1,81E+06	8,04E+06	1,39E+06
Q8BG32	*26S proteasome non-ATPase regulatory subunit 11*	6	2	47437	1,05E+06	3,06E+05	6,19E+05
P62192	*26S proteasome regulatory subunit 4*	10	2	49185	3,74E+05		6,80E+05
O88986	*2-amino-3-ketobutyrate coenzyme A ligase mitochondrial*	10	3	44931	2,22E+07		4,96E+06
Q60597	*2-oxoglutarate dehydrogenase complex component E1*	16	15	116,449	1,11E+07	1,82E+07	1,56E+07
Q99L13	*3-hydroxyisobutyrate dehydrogenase mitochondrial*	25	6	35440	3,53E+06	6,59E+06	4,57E+06
Q8BWT1	*3-ketoacyl-CoA thiolase mitochondrial*	24	8	41830	1,85E+07	1,81E+07	1,72E+07
Q921H8	*3-ketoacyl-CoA thiolase A peroxisomal*	8	3	43953	1,22E+06		1,76E+06
H3BKL5	*3-ketoacyl-CoA thiolase A peroxisomal*	9	3	38362	1,22E+06		1,76E+06
Q8VCH0	*3-ketoacyl-CoA thiolase B peroxisomal*	8	3	43995	1,22E+06		1,76E+06
Q99J99	*3-mercaptopyruvate sulfurtransferase*	17	3	33097	6,94E+05	6,28E+05	8,23E+05
F7AEH4	*40S ribosomal protein S12*	28	5	15181	9,60E+06	4,25E+06	7,95E+06

A0A1W2P7A1	*40S ribosomal protein S12*	26	5	15979	9,60E+06	4,25E+06	7,95E+06
F8WJ41	*40S ribosomal protein S15a*	43	5	12311	1,29E+07	6,26E+06	9,74E+06
A0A286YEB7	*40S ribosomal protein S24*	23	2	13605	1,34E+06	1,01E+06	1,63E+06
A0A1L1SQA8	*40S ribosomal protein S25*	46	6	10309	3,94E+07	2,36E+07	2,39E+07
A0A0G2JDW7	*40S ribosomal protein S27*	23	1	9233	1,17E+06	6,15E+05	1,34E+06
P10852	*4F2 cell-surface antigen heavy chain*	14	7	58337	6,85E+06	2,31E+06	6,65E+06
Q9JLJ2	*4-trimethylaminobutyraldehyde dehydrogenase*	8	4	53515	2,85E+06	3,54E+06	1,49E+06
P63038	*60 kDa heat shock protein mitochondrial*	28	15	60956	5,68E+07	5,74E+07	4,95E+07
P14869	*60S acidic rib*	42	10	34216	4,92E+07	2,54E+07	4,31E+07
A0A1D5RLW5	*60S ribosomal protein L18a*	48	7	17447	1,31E+07	3,59E+06	7,60E+06
Q9DCD0	*6-phosphogluconate dehydrogenase, decarboxylating*	25	11	53247	6,61E+06	2,38E+07	9,06E+06
Q9CQ60	*6-phosphogluconolactonase*	28	5	27254	1,04E+06		7,20E+05
Q8QZT1	*Acetyl-CoA acetyltransferase, mitochondrial*	35	12	44,816	4,48E+07	3,39E+07	3,55E+07
D3YYE1	*Acidic leucine-rich nuclear ph*	26	2	22954	8,51E+05	2,16E+05	1,18E+06
F6UFG6	*Acidic leucine-rich nuclear ph*	38	2	15425	8,51E+05	2,16E+05	1,18E+06
D3Z7M9	*Acidic leucine-rich nuclear ph*	22	2	26861	8,51E+05	2,16E+05	1,18E+06
O35381	*Acidic leucine-rich nuclear ph*	21	2	28538	8,51E+05	2,16E+05	1,18E+06
Q9EST5	*Acidic leucine-rich nuclear ph*	16	1	31079	7,74E+04	2,63E+05	#DIV/0!
Q91XA9	*Acidic mammalian chitinase*	30	11	52003	2,95E+07	1,14E+08	1,57E+08
Q99KI0	*Aconitate hydratase, mitochondrial*	41	30	85,464	1,09E+08	1,15E+08	9,95E+07
P68134	*Actin, alpha skeletal muscle*	75	1	42,051		1,28E+05	
P63260	*Actin, cytoplasmic 2*	76	4	41,793	1,20E+09	8,59E+08	1,00E+09
F8WGM8	*Actin, gamma, cytoplasmic 1*	85	1	11,884	4,94E+05	1,78E+05	
P63268	*Actin, gamma-enteric smooth muscle*	77	1	41,877	3,48E+05	9,42E+05	4,58E+06
A1BN54	*Actinin, alpha 1*	49	19	102,72	3,65E+07	1,46E+07	1,63E+07
P61161	*Actin-related protein 2*	14	4	44,761	2,54E+06	2,36E+06	4,20E+06
P59999	*Actin-related protein 2/3 complex subunit 4*	28	5	19667	7,83E+06	6,63E+06	5,88E+06

Q99JY9	*Actin-related protein 3*	22	5	47357	5,71E+06	1,00E+07	4,86E+06
Q91WG0	*Acylcarnitine hydrolase*	20	4	62470	3,55E+06	1,28E+06	1,34E+06
P97823	*Acyl-protein thioesterase 1*	12	2	24688	5,63E+05	5,89E+05	4,21E+05
Q9JJU8	*Adapter Sh3bgrl*	29	3	12811	2,13E+06	1,01E+06	9,79E+05
P03958	*Adenosine deaminase*	52	14	39,992	3,50E+07	3,46E+06	6,99E+07
P50247	*Adenosylhomocysteinase*	31	10	47,688	2,70E+07	9,95E+05	1,66E+07
P40124	*Adenylyl cyclase-associated protein 1*	21	8	51565	1,50E+06	3,20E+06	2,91E+06
P48962	*ADP/ATP translocase 1*	56	6	32,904	2,56E+07	1,80E+07	1,76E+07
P51881	*ADP/ATP translocase 2*	60	7	32,931	7,11E+07	6,65E+07	5,65E+07
P84078	*ADP-ribosylation factor 1*	35	2	20697	2,22E+06	6,66E+05	5,04E+06
P61205	*ADP-ribosylation factor 3*	35	2	20601	2,22E+06	6,66E+05	5,04E+06
P61750	*ADP-ribosylation factor 4*	23	1	20397	2,82E+06	3,89E+06	3,47E+06
P84084	*ADP-ribosylation factor 5*	33	2	20530	3,89E+06	3,16E+06	4,50E+06
E9Q616	*AHNAK nucleoprotein (desmoyokin)*	6	34	604264	6,78E+07	2,97E+07	3,85E+07
A0A7N9VR94	*AHNAK nucleoprotein 2*	2	4	368475	2,31E+06		3,61E+05
Q8QZR5	*Alanine aminotransferase 1*	6	3	55143	9,21E+05	7,62E+05	3,43E+05
P07724	*Albumin*	80	46	68,693	1,23E+09	1,19E+09	1,39E+09
P00329	*Alcohol dehydrogenase 1*	9	4	39771	4,82E+06	1,03E+06	1,14E+06
P28474	*Alcohol dehydrogenase class-3*	11	3	39548	1,04E+06	6,49E+05	2,15E+05
B1AV77	*Aldehyde dehydrogenase*	9	2	54330	3,73E+05		7,18E+05
B1ATI0	*Aldehyde dehydrogenase*	8	2	59277	3,73E+05		7,18E+05
P47739	*Aldehyde dehydrogenase dimeric NADP-preferring*	26	10	50481	1,34E+07	1,70E+07	1,64E+07
P47738	*Aldehyde dehydrogenase mitochondrial*	41	15	56538	3,09E+07	3,36E+07	3,12E+07
P24549	*Aldehyde dehydrogenase 1A1*	39	13	54,468	1,47E+07	1,30E+07	1,76E+07
P47740	*Aldehyde dehydrogenase family 3 member A2*	9	2	53971	3,73E+05		7,18E+05
E9Q3E1	*Aldehyde dehydrogenase family 3 member B2*	10	4	52983	2,32E+06		1,42E+06
Q9CZS1	*Aldehyde dehydrogenase X mitochondrial*	10	3	57553	3,89E+06		

Q9JII6	*Aldo-keto reductase family 1 member A1*	25	7	36587	9,20E+06	9,88E+06	7,91E+06
P45376	*Aldo-keto reductase family 1 member B1*	16	4	35732	6,25E+06	8,10E+06	4,58E+06
Q8VC28	*Aldo-keto reductase family 1 member C13*	24	2	37058	7,42E+05	3,91E+05	
F8VQM0	*Alkaline ph*	12	4	60291	1,15E+07		
F8VPQ6	*Alkaline phosphatase*	7	1	59532	1,32E+06		
Q64437	*All-trans-retinol dehydrogenase [NAD(+)] ADH7*	15	5	39904	3,08E+06	1,03E+06	2,64E+06
Q91VB8	*Alpha globin 1*	55	1	15112	9,24E+05	3,18E+06	2,04E+06
P22599	*Alpha-1-antitrypsin 1-2*	37	2	45975	7,15E+05		2,82E+05
Q00896	*Alpha-1-antitrypsin 1-3*	44	4	45823	7,41E+06	6,37E+06	7,70E+06
Q7TPR4	*Alpha-actinin-1*	49	19	103,068	3,65E+07	1,46E+07	1,63E+07
Q9JI91	*Alpha-actinin-2*	21	6	103834	3,34E+06		
P57780	*Alpha-actinin-4*	41	17	104,977	2,01E+07	1,65E+07	2,07E+07
Q9DBF1	*Alpha-aminoadipic semialdehyde dehydrogenase*	13	6	58862	4,69E+06	9,10E+05	1,74E+06
P00687	*Alpha-amylase 1*	17	1	57644	2,30E+05		
A0A494BB86	*Alpha-centractin*	16	3	37669	1,75E+06	1,60E+06	1,47E+06
P61164	*Alpha-centractin*	14	3	42614	1,75E+06	1,60E+06	1,47E+06
P17182	*Alpha-enolase*	53	15	47,141	1,62E+08	1,56E+08	1,42E+08
Q9EPC1	*Alpha-parvin*	6	2	42330	3,26E+06		
Q8VCT3	*Aminopeptidase B*	9	5	72416	2,82E+06	3,46E+06	1,71E+06
A0A0R4J1K4	*Anion exchange protein*	5	6	135437	9,12E+05	4,90E+06	3,96E+06
A0A0R4J1K9	*Anion exchange protein*	5	6	136078	9,12E+05	4,90E+06	3,96E+06
A0A0R4J101	*Anion exchange protein*	5	6	136800	9,12E+05	4,90E+06	3,96E+06
P13808	*Anion exchange protein 2*	5	6	136814	9,12E+05	4,90E+06	3,96E+06
P07146	*Anionic trypsin-2*	22	2	26204	6,60E+07		4,17E+07
F8WIT2	*Annexin*	37	20	75,289	3,89E+07	2,88E+07	2,42E+07
A0A2C9F2D2	*Annexin*	13	6	52271	4,12E+06	3,16E+06	3,17E+06
P10107	*Annexin A1*	30	9	38734	7,74E+06	9,24E+06	9,75E+06

Q9QZ10	*Annexin A10*	27	8	37,29	1,58E+07	2,87E+07	1,92E+07
P97384	*Annexin A11*	10	4	54079	1,55E+06	1,25E+06	5,87E+05
P07356	*Annexin A2*	45	15	38676	4,11E+07	3,30E+07	2,84E+07
O35639	*Annexin A3*	27	7	36384	5,31E+06	8,25E+06	6,02E+06
P97429	*Annexin A4*	34	9	35916	7,50E+06	9,18E+06	6,28E+06
P48036	*Annexin A5*	39	12	35752	4,16E+07	4,26E+07	4,02E+07
P14824	*Annexin A6*	37	20	75,885	3,89E+07	2,88E+07	2,42E+07
Q07076	*Annexin A7*	14	6	49925	4,12E+06	3,16E+06	3,17E+06
O88312	*Anterior gradient protein 2 homolog*	26	4	19920	3,94E+07	3,32E+07	2,19E+07
P32261	*Antithrombin-III*	11	5	52004	1,97E+05	1,15E+06	2,95E+06
Q5SVG5	*AP complex subunit beta*	5	4	101193	1,45E+06	1,39E+05	1,11E+06
Q5SVG4	*AP complex subunit beta*	5	4	101898	1,45E+06	1,39E+05	1,11E+06
O35643	*AP-1 complex subunit beta-1*	5	4	103935	1,45E+06	1,39E+05	1,11E+06
Q00623	*Apolipoprotein A-I*	18	5	30616	4,06E+06	7,91E+06	5,43E+06
P06728	*Apolipoprotein A-IV*	6	2	45029	1,18E+06	3,40E+05	1,46E+05
Q9Z0X1	*Apoptosis-inducing factor 1, mitochondrial*	14	7	66766	3,82E+06	5,85E+06	2,67E+06
B1AU25	*Apoptosis-inducing factor, mitochondrion-associated 1*	14	7	66114	3,82E+06	5,85E+06	2,67E+06
Q9EQX0	*Appetite-regulating hormone*	48	5	13207	2,00E+07	3,90E+07	4,27E+07
O08691	*Arginase-2 mitochondrial*	9	2	38878	1,83E+06		
Q80ZP8	*Armet protein*	19	4	19012	2,23E+07	2,64E+06	3,92E+06
Q61024	*Asparagine synthetase*	19	10	64,283	1,15E+07	6,55E+05	4,92E+06
A0A498WGK2	*Asparagine--tRNA ligase*	15	7	64208	8,96E+06		2,42E+06
Q8BP47	*Asparagine--tRNA ligase cytoplasmic*	15	7	64279	8,96E+06		2,42E+06
P05201	*Aspartate aminotransferase cytoplasmic*	25	8	46248	7,00E+06	1,04E+07	9,75E+06
P05202	*Aspartate aminotransferase, mitochondrial*	32	12	47,411	2,43E+07	3,22E+07	3,35E+07
Q922B2	*Aspartate--tRNA ligase cytoplasmic*	8	4	57147	4,20E+06		3,50E+05
Q9Z2W0	*Aspartyl aminopeptidase*	20	7	52207	5,98E+06	1,97E+06	2,00E+06

Q99MQ4	*Asporin*	17	5	42573	1,98E+06	7,93E+05	9,36E+05
Q9CQQ7	*ATP synthase F(0) complex subunit B1 mitochondrial*	12	4	28949	8,12E+06	1,40E+07	9,05E+06
Q78IK2	*ATP synthase membrane subunit K mitochondrial*	43	2	6382	6,90E+06	6,77E+06	6,01E+06
P03930	*ATP synthase protein 8*	43	3	7766	4,04E+06	4,75E+06	4,01E+06
Q03265	*ATP synthase subunit alpha mitochondrial*	52	25	59753	2,89E+08	3,37E+08	3,15E+08
P56480	*ATP synthase subunit beta mitochondrial*	64	23	56301	3,06E+08	2,67E+08	2,73E+08
Q9DCX2	*ATP synthase subunit d, mitochondria*	71	11	18,749	1,97E+07	1,98E+07	1,43E+07
Q9D3D9	*ATP synthase subunit delta mitochondrial*	17	3	17600	2,53E+07	2,71E+07	1,54E+07
Q06185	*ATP synthase subunit e mitochondrial*	61	4	8236	1,09E+07	1,51E+07	9,33E+06
F8WHP8	*ATP synthase subunit f mitochondrial*	16	1	9006	1,89E+07	2,06E+07	1,91E+07
P56135	*ATP synthase subunit f mitochondrial*	14	1	10344	1,89E+07	2,06E+07	1,91E+07
Q8C2Q8	*ATP synthase subunit gamma*	27	7	30256	1,74E+07	2,38E+07	1,23E+07
Q91VR2	*ATP synthase subunit gamma mitochondrial*	25	7	32886	1,74E+07	2,38E+07	1,23E+07
Q9DB20	*ATP synthase subunit O, mitochondrial*	63	10	23,364	2,67E+07	2,99E+07	2,67E+07
P97450	*ATP synthase-coupling factor 6 mitochondrial*	42	4	12496	4,51E+06	8,94E+06	1,24E+07
Q91V92	*ATP-citrate synthase*	4	4	119728	1,69E+06	5,56E+05	6,86E+05
Q3V117	*ATP-citrate synthase*	4	4	120795	1,69E+06	5,56E+05	6,86E+05
Q8C605	*ATP-dependent 6-ph*	4	2	85547	1,00E+06	1,82E+05	3,73E+05
Q9WUA3	*ATP-dependent 6-ph*	4	2	85455	1,00E+06	1,82E+05	3,73E+05
P12382	*ATP-dependent 6-ph*	6	3	85360	6,08E+05	1,91E+05	5,49E+05
Q91VR5	*ATP-dependent RNA helicase DDX1*	6	3	82500	2,78E+05	3,98E+05	1,72E+05
Q8VDW0	*ATP-dependent RNA helicase DDX39A*	6	3	49067	2,64E+06	4,25E+05	2,22E+06
Q62167	*ATP-dependent RNA helicase DDX3X*	18	10	73102	4,73E+06	5,46E+06	5,22E+06
B1B0C7	*Basement membrane-specific heparan sulfate proteoglycan core protein*	9	38	469027	3,40E+07	1,71E+07	1,88E+07
E9PZ16	*Basement membrane-specific heparan sulfate proteoglycan core protein*	9	38	469789	3,40E+07	1,71E+07	1,88E+07
K3W4Q8	*Basigin*	25	5	24116	3,83E+06	6,39E+06	2,92E+06
P18572	*Basigin*	14	5	42445	3,83E+06	6,39E+06	2,92E+06

J3QP71	*Basigin (Fragment)*	27	5	21721	3,83E+06	6,39E+06	2,92E+06
Q8BFZ3	*Beta-actin-like protein 2*	34	2	42,004		4,04E+04	3,08E+06
P21550	*Beta-enolase*	17	3	47025	2,86E+06		
A8DUK4	*Beta-globin*	89	2	15,748	1,68E+06	1,43E+06	
A0A494B923	*Bifunctional 3'-ph*	18	9	69485	3,90E+07	1,92E+06	1,12E+06
A0A494BB18	*Bifunctional 3'-ph*	18	9	69842	3,90E+07	1,92E+06	1,12E+06
O88428	*Bifunctional 3'-ph*	18	9	70351	3,90E+07	1,92E+06	1,12E+06
Q8CGC7	*Bifunctional glutamate/proline--tRNA ligase*	8	7	170078	2,01E+06	1,15E+06	1,61E+06
P28653	*Biglycan*	22	7	41,639	3,76E+06	1,11E+06	1,71E+06
Q64285	*Bile salt-activated lipase*	23	13	65,813	8,85E+07		6,51E+07
O88374	*Branched-chain-amino-acid aminotransferase*	17	5	39774	2,43E+06	3,89E+06	5,02E+06
O35855	*Branched-chain-amino-acid aminotransferase mitochondrial*	15	5	44127	2,43E+06	3,89E+06	5,02E+06
Q922D8	*C-1-tetrahydrofolate synthase cytoplasmic*	9	7	101200	5,97E+06	1,91E+06	5,38E+06
P09803	*Cadherin-1*	4	3	98256	1,93E+06	3,11E+06	1,68E+06
A0A0R4IZW5	*Cadherin-1*	4	3	98284	1,93E+06	3,11E+06	1,68E+06
B1ATS4	*Calcium-transporting ATPase*	6	1	107600	5,58E+05		
B1ATS5	*Calcium-transporting ATPase*	6	1	111709	5,58E+05		
E9Q559	*Calcium-transporting ATPase*	6	1	112504	5,58E+05		
E9QA15	*Caldesmon 1*	29	6	89274	2,44E+07	2,67E+07	2,19E+07
P0DP26	*Calmodulin-1*	34	6	16838	4,77E+07	5,33E+07	4,94E+07
P0DP27	*Calmodulin-2*	34	6	16838	4,77E+07	5,33E+07	4,94E+07
P0DP28	*Calmodulin-3*	34	6	16838	4,77E+07	5,33E+07	4,94E+07
P35564	*Calnexin*	18	12	67278	2,15E+07	8,82E+06	8,92E+06
A0A0G2JDV8	*Calponin*	14	2	31395	1,78E+07	1,21E+07	1,47E+07
Q08091	*Calponin-1*	65	14	33,356	3,12E+08	1,16E+08	2,15E+08
Q9DAW9	*Calponin-3*	12	2	36429	1,78E+07	1,21E+07	1,47E+07
P14211	*Calreticulin*	41	17	47,995	7,08E+07	3,35E+07	4,71E+07

Q6XLQ8	*Calumenin*	17	5	37118	3,21E+06	1,08E+06	2,02E+06
P00920	*Carbonic anhydrase 2*	38	8	29033	4,37E+07	6,76E+07	4,37E+07
P16015	*Carbonic anhydrase 3*	32	6	29366	8,00E+06	1,67E+07	
Q8VHB5	*Carbonic anhydrase 9*	7	3	47265	2,58E+05	6,02E+05	4,66E+05
P48758	*Carbonyl reductase [NADPH] 1*	51	10	30641	1,50E+07	2,22E+07	1,54E+07
Q8K354	*Carbonyl reductase [NADPH] 3*	32	6	30953	1,05E+06	4,94E+06	3,31E+06
P23953	*Carboxylesterase 1C*	7	1	61056		1,97E+05	3,76E+05
Q8VCT4	*Carboxylesterase 1D*	12	4	61788	1,29E+06	5,99E+05	3,07E+05
Q7TPZ8	*Carboxypeptidase A1*	21	8	47385	1,01E+08		9,04E+07
Q504N0	*Carboxypeptidase A2*	13	5	47057	7,89E+06	2,51E+06	1,39E+07
B2RS76	*carboxypeptidase B1 (Tissue)*	46	14	47574	1,11E+08		1,42E+08
O08738	*Caspase-6*	11	3	31595	4,48E+05	1,12E+06	2,63E+05
P24270	*Catalase*	15	6	59795	6,74E+06	3,00E+06	9,40E+05
P26231	*Catenin alpha-1*	8	4	100106	1,33E+06	1,89E+06	7,48E+05
A0A494BAD0	*Catenin alpha-1 (Fragment)*	17	4	45752	1,33E+06	1,89E+06	7,48E+05
G3X9V2	*Catenin delta-1*	2	1	104104	8,61E+05	6,76E+05	9,32E+05
P30999	*Catenin delta-1*	2	1	104925	8,61E+05	6,76E+05	9,32E+05
E9Q8Z5	*Catenin delta-1*	2	1	104995	8,61E+05	6,76E+05	9,32E+05
E9Q8Z6	*Catenin delta-1*	2	1	107354	8,61E+05	6,76E+05	9,32E+05
E9Q8Z8	*Catenin delta-1*	2	1	108175	8,61E+05	6,76E+05	9,32E+05
E9Q986	*Catenin delta-1*	2	1	92478	8,61E+05	6,76E+05	9,32E+05
D3Z7H6	*Catenin delta-1*	2	1	93427	8,61E+05	6,76E+05	9,32E+05
E9Q906	*Catenin delta-1*	2	1	93497	8,61E+05	6,76E+05	9,32E+05
E9Q907	*Catenin delta-1*	2	1	95857	8,61E+05	6,76E+05	9,32E+05
E9Q905	*Catenin delta-1*	2	1	96677	8,61E+05	6,76E+05	9,32E+05
E9Q8Z4	*Catenin delta-1*	2	1	96872	8,61E+05	6,76E+05	9,32E+05
E9Q8Z9	*Catenin delta-1*	2	1	98007	8,61E+05	6,76E+05	9,32E+05

D3Z2H2	*Catenin delta-1*	2	1	98828	8,61E+05	6,76E+05	9,32E+05
E9Q903	*Catenin delta-1*	2	1	99719	8,61E+05	6,76E+05	9,32E+05
E9Q901	*Catenin delta-1*	2	1	101258	8,61E+05	6,76E+05	9,32E+05
E9Q904	*Catenin delta-1*	2	1	102078	8,61E+05	6,76E+05	9,32E+05
F8WIR1	*Cathepsin D*	24	7	44,215	3,94E+06	5,05E+06	5,04E+06
P18242	*Cathepsin D*	23	7	44,954	3,94E+06	5,05E+06	5,04E+06
O54724	*Caveolae-associated protein 1*	32	13	43954	2,78E+07	1,54E+07	1,11E+07
Q63918	*Caveolae-associated protein 2*	7	3	46764	2,11E+07	1,98E+07	5,76E+05
P49817	*Caveolin-1*	54	10	20539	2,42E+07	4,55E+06	7,70E+06
P35762	*CD81 antigen*	10	1	25815	2,41E+06	1,52E+06	1,61E+06
A0A140LJL0	*CD81 antigen (Fragment)*	19	1	13176	2,41E+06	1,52E+06	1,61E+06
P40240	*CD9 antigen*	14	2	25,258	4,37E+06		2,13E+06
P60766	*Cell division control protein 42 homolog*	18	3	21259	3,74E+06	2,86E+06	3,26E+06
Q8R2Y2	*Cell surface glycoprotein MUC18*	7	3	71546	1,31E+06		8,79E+05
Q52KG9	*Chaperonin containing Tcp1 subunit 6a (Zeta)*	16	8	58076	5,90E+06	3,69E+06	3,32E+06
Q9Z1Q5	*Chloride intracellular channel protein 1*	36	6	27013	5,55E+06	1,03E+07	8,04E+06
Q8BHB9	*Chloride intracellular channel protein 6*	41	16	62,886	2,77E+07	5,29E+07	2,92E+07
Q9ER05	*Chymopasin*	30	5	28135	5,53E+07		6,99E+07
B7ZWD6	*Chymosin*	5	2	42511	8,43E+05	8,52E+05	2,09E+06
Q91X79	*Chymotrypsin-like elastase family member 1*	36	6	28,901	2,33E+07		2,90E+07
P05208	*Chymotrypsin-like elastase family member 2A*	44	7	28,914	1,06E+08		1,06E+08
Q9CQ52	*Chymotrypsin-like elastase family member 3B*	35	5	28905	8,96E+07		8,97E+07
Q9CR35	*Chymotrypsinogen B*	37	8	27,822	3,05E+08		3,06E+08
Q80X68	*Citrate synthase*	12	1	52325	1,37E+06		2,22E+05
Q9CZU6	*Citrate synthase mitochondrial*	25	7	51737	2,58E+07	2,77E+07	1,69E+07
Q5SXR6	*Clathrin heavy chain*	23	35	191,986	2,21E+07	2,02E+07	2,18E+07
Q68FD5	*Clathrin heavy chain 1*	23	35	191,557	2,21E+07	2,02E+07	2,18E+07

P56857	*Claudin-18*	8	2	28122	1,57E+06	3,04E+06	4,11E+06
Q06890	*Clusterin*	10	4	51656	2,24E+06	3,01E+06	3,48E+06
Q9CQI6	*Coact*	16	3	15944	5,07E+06	7,31E+06	5,30E+06
Q8CIE6	*Coatomer subunit alpha*	12	14	138432	7,22E+06	1,04E+06	3,27E+06
F8WHL2	*Coatomer subunit alpha*	12	14	139410	7,22E+06	1,04E+06	3,27E+06
Q9JIF7	*Coatomer subunit beta*	7	5	107066	3,05E+06	6,00E+05	2,59E+06
O55029	*Coatomer subunit beta'*	9	7	102449	4,83E+06	1,92E+06	3,92E+06
Q5XJY5	*Coatomer subunit delta*	17	8	57230	8,83E+06	2,15E+06	4,94E+06
Q9QZE5	*Coatomer subunit gamma-1*	9	5	97513	2,26E+06	1,73E+06	2,19E+06
Q9QXK3	*Coatomer subunit gamma-2*	3	1	97680	#DIV/0!		6,56E+06
P52787	*Cobalamin binding intrinsic factor*	18	5	45497	8,49E+06	1,26E+07	1,25E+07
P18760	*Cofilin-1*	60	8	18,56	1,34E+07	9,67E+06	7,54E+06
P45591	*Cofilin-2*	28	2	18710	3,57E+05		8,00E+04
Q9D9P1	*Coiled-coil-helix-coiled-coil-helix domain containing 3*	35	6	20376	6,47E+06	5,90E+06	4,40E+06
D3Z0L4	*Coiled-coil-helix-coiled-coil-helix domain containing 4*	32	6	22452	6,47E+06	5,90E+06	4,40E+06
Q9CQC2	*Colipase*	50	5	12,445	3,55E+07	9,69E+07	9,86E+07
J3QQ16	*Collagen type VI alpha 3*	27	60	288693	1,78E+08	5,90E+07	9,55E+07
A0A087WS16	*Collagen type VI alpha 3*	27	60	288691	1,78E+08	5,90E+07	9,55E+07
P11087	*Collagen alpha-1(I) chain*	6	7	138033	4,06E+08	2,79E+08	3,88E+08
P08121	*Collagen alpha-1(III) chain*	2	3	138944	1,75E+07	2,71E+06	5,37E+06
P02463	*Collagen alpha-1(IV) chain*	2	3	160679	4,46E+06	3,42E+06	2,45E+06
Q04857	*Collagen alpha-1(VI) chain*	15	13	108489	4,19E+07	1,80E+07	2,38E+07
E9PX70	*Collagen alpha-1(XII) chain*	3	7	333731	1,63E+06	6,98E+05	6,66E+05
Q60847	*Collagen alpha-1(XII) chain*	3	7	340215	1,63E+06	6,98E+05	6,66E+05
B7ZNH7	*Collagen alpha-1(XIV) chain*	3	4	192753	1,41E+06	2,87E+05	7,70E+05
Q80X19	*Collagen alpha-1(XIV) chain*	3	4	193011	1,41E+06	2,87E+05	7,70E+05
K3W4R4	*Collagen alpha-1(XIV) chain*	3	4	193139	1,41E+06	2,87E+05	7,70E+05

Q01149	*Collagen alpha-2(I) chain*	8	9	129557	5,20E+08	3,76E+08	5,04E+08
P08122	*Collagen alpha-2(IV) chain*	4	5	167,325	4,02E+06	5,66E+06	6,36E+06
Q02788	*Collagen alpha-2(VI) chain*	18	18	110334	6,22E+07	2,12E+07	3,24E+07
A0A140T8T7	*Collagen alpha-5(VI) chain*	3	6	289514	4,49E+05	1,38E+06	3,36E+05
A6H584	*Collagen alpha-5(VI) chain*	3	6	289572	4,49E+05	1,38E+06	3,36E+05
A0A140T8W1	*Collagen alpha-5(VI) chain*	3	6	289613	4,49E+05	1,38E+06	3,36E+05
P01027	*Complement C3*	4	5	186483	3,68E+05	6,59E+05	8,32E+05
Q8JZN5	*Complex I assembly factor ACAD9 mitochondrial*	6	3	68722	6,46E+05	3,60E+05	3,32E+05
Q62266	*Cornifin-A*	37	5	15765	4,14E+07	9,63E+06	6,95E+07
Q9WUM4	*Coronin-1C*	24	10	53121	7,46E+06	4,01E+06	4,95E+06
Q04447	*Creatine kinase B-type*	66	17	42,713	9,80E+07	9,91E+07	8,69E+07
P07310	*Creatine kinase M-type*	34	9	43045	2,15E+07	2,18E+06	2,05E+06
P30275	*Creatine kinase U-type, mitochondrial*	33	10	47,004	2,70E+07	4,60E+07	3,64E+07
Q6ZQ38	*Cullin-associated NEDD8-dissociated protein 1*	3	3	136331	2,73E+05	1,12E+05	4,94E+05
Q8VCN5	*Cystathionine gamma-lyase*	13	5	43567	7,23E+06	#DIV/0!	
P97315	*Cysteine and glycine-rich protein 1*	64	11	20,583	1,38E+08	7,32E+07	9,96E+07
G5E850	*Cytochrome b5*	56	4	11142	3,50E+06	5,24E+06	4,28E+06
P56395	*Cytochrome b5*	41	4	15241	3,50E+06	5,24E+06	4,28E+06
Q9CQX2	*Cytochrome b5 type B*	32	2	16318	8,26E+06	6,44E+06	9,09E+06
Q9CZ13	*Cytochrome b-c1 complex subunit 1 mitochondrial*	29	11	52852	2,41E+07	1,34E+07	2,57E+07
Q9DB77	*Cytochrome b-c1 complex subunit 2, mitochondrial*	37	13	48,235	3,30E+07	3,07E+07	2,90E+07
P99028	*Cytochrome b-c1 complex subunit 6 mitochondrial*	48	4	10435	6,35E+06	4,51E+06	7,03E+06
Q9D855	*Cytochrome b-c1 complex subunit 7*	22	2	13527	3,34E+06	4,76E+06	7,41E+06
Q9CQB4	*Cytochrome b-c1 complex subunit 7*	22	2	13561	3,34E+06	4,76E+06	7,41E+06
Q9CR68	*Cytochrome b-c1 complex subunit Rieske mitochondrial*	34	9	29368	1,33E+07	2,66E+07	3,22E+07
P62897	*Cytochrome c somatic*	55	7	11605	2,78E+07	2,48E+07	2,16E+07
P00405	*Cytochrome c oxidase subunit 2*	19	4	25976	5,27E+07	5,34E+07	5,48E+07

P19783	*Cytochrome c oxidase subunit 4 isoform 1 mitochondrial*	48	7	19530	3,47E+07	4,17E+07	3,52E+07
P12787	*Cytochrome c oxidase subunit 5A mitochondrial*	21	3	16101	2,21E+07	2,58E+07	2,37E+07
P19536	*Cytochrome c oxidase subunit 5B mitochondrial*	30	4	13813	1,62E+07	1,94E+07	1,37E+07
Q9D881	*Cytochrome c oxidase subunit 5B mitochondrial*	30	4	13847	1,62E+07	1,94E+07	1,37E+07
P56391	*Cytochrome c oxidase subunit 6B1*	70	6	10071	3,25E+07	6,24E+07	4,37E+07
Q9CPQ1	*Cytochrome c oxidase subunit 6C*	29	3	8469	3,33E+07	4,13E+07	3,45E+07
Q62425	*Cytochrome c oxidase subunit NDUFA4*	48	5	9327	5,26E+07	6,17E+07	5,70E+07
A0A0N4SVQ1	*Cytochrome c oxidase subunit NDUFA4*	80	5	5877	5,26E+07	6,17E+07	5,70E+07
Q9D0M3	*Cytochrome c1 heme protein mitochondrial*	18	5	35328	2,36E+06	1,35E+06	2,02E+06
D3Z7F1	*Cytochrome P450*	15	5	49792	3,73E+06	5,87E+06	9,24E+06
Q148B1	*Cytochrome P450 family 2 subfamily c polypeptide 65*	7	3	56211	7,11E+05	3,39E+05	5,79E+05
Q9DBX6	*Cytochrome P450 2S1*	13	5	55632	3,73E+06	5,87E+06	9,24E+06
P28271	*Cytoplasmic aconitate hydratase*	8	6	98126	4,19E+06	3,01E+06	8,51E+06
Q9JHU4	*Cytoplasmic dynein 1 heavy chain 1*	5	20	532050	5,71E+06	6,08E+06	4,58E+06
Q8R0Y6	*Cytosolic 10-formyltetrahydrofolate dehydrogenase*	9	4	98709	1,04E+06	1,48E+06	1,50E+06
Q9D1A2	*Cytosolic non-specific dipeptidase*	8	3	52767	1,41E+06	5,33E+05	1,00E+06
Q61753	*D-3-phosphoglycerate dehydrogenase*	20	9	56586	1,61E+07		1,68E+07
Q80XN0	*D-beta-hydroxybutyrate dehydrogenase mitochondrial*	10	3	38299	3,11E+06	1,10E+06	2,85E+06
O35215	*D-dopachrome decarboxylase*	23	1	13077	2,26E+06	2,53E+06	2,38E+06
P28654	*Decorin*	31	10	39809	1,69E+07	8,69E+06	1,36E+07
Q9Z110	*Delta-1-pyrroline-5-carboxylate synthase*	8	6	87266	3,67E+06		6,88E+04
P10518	*Delta-aminolevulinic acid dehydratase*	29	7	36,024	7,02E+06	6,00E+06	8,31E+06
E9QLW7	*Dermokine*	8	4	51056	3,76E+06		8,68E+06
E9QQ29	*Dermokine*	8	4	52144	3,76E+06		8,68E+06
Q6P253	*Dermokine*	8	4	51661	3,76E+06		8,68E+06
E9Q2P1	*Dermokine*	8	4	52812	3,76E+06		8,68E+06
P31001	*Desmin*	67	27	53,498	7,46E+08	4,22E+08	5,63E+08

E9Q557	*Desmoplakin*	8	25	332913	6,35E+06	9,36E+05	1,64E+07
Q9R0P5	*Destrin*	79	14	18,522	7,06E+07	5,48E+07	5,12E+07
O08749	*Dihydrolipoyl dehydrogenase, mitochondrial*	23	9	54,272	1,56E+07	1,28E+07	9,28E+06
Q8BMF4	*Dihydrolipoyllysine-residue acetyltransferase component*	19	10	67,942	9,73E+06	8,40E+06	7,69E+06
Q9D2G2	*Dihydrolipoyllysine-residue succinyltransferase component*	17	7	48995	1,26E+07	1,29E+07	1,24E+07
O08553	*Dihydropyrimidinase-related protein 2*	6	1	62278	2,77E+06	1,87E+06	1,54E+06
Q3TT92	*Dihydropyrimidinase-related protein 3*	6	1	61780	2,73E+06	3,84E+06	1,23E+06
Q62188	*Dihydropyrimidinase-related protein 3*	6	1	61936	2,73E+06	3,84E+06	1,23E+06
E9PWE8	*Dihydropyrimidinase-related protein 3*	5	1	73884	2,73E+06	3,84E+06	1,23E+06
Q99KV1	*DnaJ homolog subfamily B member 11*	7	2	40555	4,50E+06	1,03E+06	1,85E+06
A2ACG7	*Dolichyl-diph*	26	12	67502	1,29E+07	5,11E+06	8,98E+06
Q9DBG6	*Dolichyl-diph*	25	12	69063	1,29E+07	5,11E+06	8,98E+06
Q91YQ5	*Dolichyl-diph*	31	17	68528	2,67E+07	1,86E+07	2,65E+07
O54734	*Dolichyl-diph*	17	8	49028	1,75E+07	9,25E+06	1,19E+07
P61804	*Dolichyl-diph*	27	3	12497	4,38E+06	3,58E+06	3,23E+06
P46978	*Dolichyl-diphosphooligosaccharide--protein glycosyltransferase*	10	7	80598	1,39E+07	2,64E+06	7,35E+06
Q62418	*Drebrin-like protein*	7	2	48700	7,62E+05	7,46E+05	8,97E+05
A0A0J9YUE9	*Dynamin GTPase*	4	4	94033	6,62E+05	3,88E+05	5,59E+05
A0A0J9YUN4	*Dynamin GTPase*	4	4	97295	6,62E+05	3,88E+05	5,59E+05
Q3T9X3	*Dynamin GTPase*	4	4	97195	6,62E+05	3,88E+05	5,59E+05
Q3TCR7	*Dynamin GTPase*	4	4	97987	6,62E+05	3,88E+05	5,59E+05
F8WIV5	*Dynamin GTPase*	4	4	98097	6,62E+05	3,88E+05	5,59E+05
G3X9G4	*Dynamin GTPase*	4	4	98074	6,62E+05	3,88E+05	5,59E+05
P39053	*Dynamin-1*	4	4	97803	6,62E+05	3,88E+05	5,59E+05
P39054	*Dynamin-2*	4	4	98145	6,62E+05	3,88E+05	5,59E+05
Q8CCJ3	*E3 UFM1-protein ligase 1*	6	4	89520	7,86E+05		8,17E+05
Q9WVK4	*EH domain-containing protein 1*	9	3	60603	#DIV/0!	3,10E+05	5,13E+04

Q8BH64	*EH domain-containing protein 2*	27	12	61175	1,19E+07	2,89E+06	3,20E+06
Q9EQP2	*EH domain-containing protein 4*	7	4	61481	1,16E+06	1,14E+06	4,33E+05
P54320	*Elastin*	5	3	71938	1,60E+07	7,67E+06	8,45E+06
Q8BH59	*Electrogenic aspartate/glutamate antiporter SLC25A12 mitochondrial*	16	5	74570	7,66E+05	1,37E+06	1,86E+06
Q9QXX4	*Electrogenic aspartate/glutamate antiporter SLC25A13 mitochondrial*	6	1	74467	#DIV/0!	3,57E+05	
Q99LC5	*Electron transfer flavoprotein subunit alpha, mitochondrial*	46	12	35,009	2,21E+07	1,68E+07	1,47E+07
Q9DCW4	*Electron transfer flavoprotein subunit beta*	47	11	27,623	1,94E+07	2,41E+07	2,38E+07
P10126	*Elongation factor 1-alpha 1*	41	16	50,114	5,09E+08	3,45E+08	3,86E+08
O70251	*Elongation factor 1-beta*	44	7	24694	1,88E+07	7,45E+06	1,24E+07
P57776	*Elongation factor 1-delta*	20	4	31293	3,37E+06	3,50E+06	5,07E+06
Q80T06	*Elongation factor 1-delta*	20	4	31292	3,37E+06	3,50E+06	5,07E+06
A0A0R4J1E2	*Elongation factor 1-delta*	8	4	72930	3,37E+06	3,50E+06	5,07E+06
E9QN08	*Elongation factor 1-delta (Fragment)*	22	4	27218	3,37E+06	3,50E+06	5,07E+06
Q9D8N0	*Elongation factor 1-gamma*	22	12	50061	2,19E+07	4,61E+06	1,51E+07
P58252	*Elongation factor 2*	37	29	95,314	1,14E+08	5,27E+07	1,07E+08
Q8BFR5	*Elongation factor Tu, mitochondrial*	27	10	49,508	1,20E+07	1,17E+07	1,09E+07
P20029	*Endoplasmic reticulum chaperone BiP*	50	29	72422	2,90E+08	1,39E+08	2,26E+08
P57759	*Endoplasmic reticulum resident protein 29*	16	4	28823	2,56E+06	6,57E+05	2,72E+06
Q9D1Q6	*Endoplasmic reticulum resident protein 44*	20	6	46853	4,13E+06	8,77E+05	2,46E+06
P08113	*Endoplasmin*	34	26	92476	8,67E+07	4,70E+07	7,54E+07
P42125	*Enoyl-CoA delta isomerase 1 mitochondrial*	18	5	32250	5,44E+06	6,52E+06	5,51E+06
A0A452J8A5	*Enoyl-CoA delta isomerase 1 mitochondrial*	19	5	32023	5,44E+06	6,52E+06	5,51E+06
A0A3Q4EC00	*Enoyl-CoA delta isomerase 1 mitochondrial*	21	5	27842	5,44E+06	6,52E+06	5,51E+06
Q8BH95	*Enoyl-CoA hydratase, mitochondrial*	26	6	31,474	5,85E+06	1,22E+07	8,33E+06
Q6YI28	*EP1*	15	3	23675	1,41E+06	2,81E+07	9,48E+06
A0A7N9VRC4	*Epiplakin*	3	5	381693	5,23E+06		3,75E+06
Q8R0W0	*Epiplakin*	1	5	724747	5,23E+06		3,75E+06

Q8BFZ9	*Erlin-2*	9	3	37873	8,15E+05	8,34E+05	4,23E+05
A0A1Y7VNF4	*ERO1-like protein beta*	12	5	55895	3,14E+06		1,04E+06
Q8R2E9	*ERO1-like protein beta*	13	5	53518	3,14E+06		1,04E+06
A0A1Y7VJM4	*ERO1-like protein beta*	12	5	55260	3,14E+06		1,04E+06
Q9EQ06	*Estradiol 17-beta-dehydrogenase 11*	20	4	32881	1,42E+06	4,70E+06	1,12E+06
P60843	*Eukaryotic initiation factor 4A-I*	27	5	46154	3,10E+06	1,21E+06	3,40E+06
P10630	*Eukaryotic initiation factor 4A-II*	21	1	46402	1,50E+05		
Q8BWY3	*Eukaryotic peptide chain release factor subunit 1*	8	3	49031	1,03E+06	1,68E+05	
Q99L45	*Eukaryotic translation initiation factor 2 subunit 2*	20	5	38092	2,05E+06	8,24E+05	1,04E+06
P23116	*Eukaryotic translation initiation factor 3 subunit A*	6	8	161936	1,62E+06	1,18E+06	1,88E+06
Q8JZQ9	*Eukaryotic translation initiation factor 3 subunit B*	6	4	91370	1,61E+06	8,26E+05	4,44E+05
Q9Z1D1	*Eukaryotic translation initiation factor 3 subunit G*	12	3	35638	1,86E+06	2,14E+06	2,36E+06
Q91WK2	*Eukaryotic translation initiation factor 3 subunit H*	8	2	39832	1,45E+06		2,06E+05
Q8QZY1	*Eukaryotic translation initiation factor 3 subunit L*	4	2	66613	1,24E+06	1,47E+06	1,28E+06
E9PVC6	*Eukaryotic translation initiation factor 4 gamma 1*	2	3	171283	2,02E+06	5,22E+05	1,45E+06
A0A0J9YUS5	*Eukaryotic translation initiation factor 4 gamma 1*	2	3	145312	2,02E+06	5,22E+05	1,45E+06
E9Q9E1	*Eukaryotic translation initiation factor 4 gamma 1*	2	3	175330	2,02E+06	5,22E+05	1,45E+06
Q6NZJ6	*Eukaryotic translation initiation factor 4 gamma 1*	2	3	176076	2,02E+06	5,22E+05	1,45E+06
E9PVC5	*Eukaryotic translation initiation factor 4 gamma 1*	2	3	175458	2,02E+06	5,22E+05	1,45E+06
A0A0A0MQM0	*Eukaryotic translation initiation factor 5A (Fragment)*	21	4	16303	1,61E+07	6,42E+05	8,73E+06
P63242	*Eukaryotic translation initiation factor 5A-1*	21	4	16832	1,61E+07	6,42E+05	8,73E+06
Q3U7R1	*Extended synaptotagmin-1*	7	6	121553	8,65E+05	6,45E+05	6,56E+05
P26040	*Ezrin*	29	11	69407	2,62E+07	4,07E+07	2,73E+07
Q5RKN9	*F-actin-capping protein subunit alpha*	15	2	32954	5,37E+05	8,23E+05	1,06E+06
P47753	*F-actin-capping protein subunit alpha-1*	15	2	32940	5,37E+05	8,23E+05	1,06E+06
P47754	*F-actin-capping protein subunit alpha-2*	22	3	32967	1,77E+06	1,08E+06	1,83E+06
Q920E5	*Farnesyl pyroph*	7	2	40582	1,01E+06	3,83E+05	1,10E+06

A0A0G2JDJ5	*Farnesyl pyroph*	20	2	13875	1,01E+06	3,83E+05	1,10E+06
A0A0G2JEB3	*Farnesyl pyroph*	11	2	25945	1,01E+06	3,83E+05	1,10E+06
A0A0U1RNJ1	*Fatty acid synthase*	8	18	272,228	5,23E+06	8,56E+06	1,94E+06
P19096	*Fatty acid synthase*	8	18	272,428	5,23E+06	8,56E+06	1,94E+06
Q05816	*Fatty acid-binding protein 5*	48	6	15,137	5,76E+07	1,70E+07	5,71E+07
P04117	*Fatty acid-binding protein, adipocyte*	38	6	14,65	1,40E+07	1,45E+07	7,32E+06
E9Q9C6	*Fc fragment of IgG-binding protein*	2	4	275239	3,30E+06	2,30E+06	3,58E+06
Q8CIB5	*Fermitin family homolog 2*	11	7	77800	1,11E+07	3,61E+06	5,45E+06
Q9CPX4	*Ferritin*	13	2	20772	2,23E+06	1,88E+06	1,31E+06
P29391	*Ferritin light chain 1*	13	2	20802	2,23E+06	1,88E+06	1,31E+06
Q61554	*Fibrillin-1*	7	19	312,298	1,82E+07	8,58E+06	1,78E+07
A0A087WS56	*Fibronectin*	6	11	249550	6,94E+06	2,78E+06	6,69E+06
Q3UHL6	*Fibronectin*	6	11	260126	6,94E+06	2,78E+06	6,69E+06
A0A087WR50	*Fibronectin*	6	11	262840	6,94E+06	2,78E+06	6,69E+06
P11276	*Fibronectin*	6	11	272536	6,94E+06	2,78E+06	6,69E+06
A0A0A6YVU7	*Filaggrin*	29	4	26670	3,60E+07	8,86E+06	1,30E+08
A0A0A6YXH0	*Filaggrin*	28	4	27106	3,60E+07	8,86E+06	1,30E+08
A0A0A6YY62	*Filaggrin*	21	4	35580	3,60E+07	8,86E+06	1,30E+08
A0A0A6YXI5	*Filaggrin*	17	3	40397	1,52E+06		8,31E+05
B7FAU9	*Filamin, alpha*	56	105	280501	1,14E+09	7,38E+08	7,90E+08
Q8BTM8	*Filamin-A*	56	105	281222	1,14E+09	7,38E+08	7,90E+08
Q80X90	*Filamin-B*	10	14	277822	6,41E+06	4,91E+06	2,17E+06
Q8VHX6	*Filamin-C*	28	50	291117	6,13E+07	1,04E+07	4,01E+07
P97447	*Four and a half LIM domains protein 1*	42	11	31889	4,53E+07	1,94E+07	2,36E+07
A2AEX8	*Four and a half LIM domains protein 1*	40	11	33564	4,53E+07	1,94E+07	2,36E+07
A2AEX6	*Four and a half LIM domains protein 1*	39	11	35081	4,53E+07	1,94E+07	2,36E+07
Q53YU8	*Foveolin*	40	7	20,134	1,08E+08	3,77E+08	2,51E+08

P70695	*Fructose-1,6-bisphosphatase isozyme 2*	15	4	36947	8,28E+06		
P05064	*Fructose-bisphosphate aldolase A*	42	8	39,356	3,55E+07	2,72E+07	1,95E+07
Q91Y97	*Fructose-bisphosphate aldolase B*	22	7	39,507	2,18E+07		
P97807	*Fumarate hydratase mitochondrial*	41	17	54357	2,46E+07	1,93E+07	2,06E+07
Q9R0N0	*Galactokinase*	10	4	42295	9,84E+05	1,11E+06	1,52E+06
Q9CRB1	*Galectin*	68	7	15202	9,89E+07	3,61E+06	9,46E+07
P16045	*Galectin-1*	47	6	14866	2,69E+07	9,95E+06	1,04E+07
Q9CQW5	*Galectin-2*	66	8	14,88	2,13E+07	2,10E+08	3,27E+07
Q9EST1	*Gasdermin-A*	11	1	49593			1,12E+06
Q32M21	*Gasdermin-A2*	12	1	49844	1,96E+06	3,74E+06	2,61E+06
Q9D7R7	*Gastricsin*	7	3	42849	3,81E+07	5,90E+07	6,73E+07
Q9CR36	*Gastrokine-1*	40	7	20,134	1,08E+08	3,77E+08	2,51E+08
Q9CQS6	*Gastrokine-2*	38	8	20,469	7,74E+07	2,41E+08	1,78E+08
Q8K0C9	*GDP-mann*	12	4	41985	3,42E+06	3,20E+06	3,52E+06
P13020	*Gelsolin*	23	13	85942	2,49E+07	3,25E+07	2,50E+07
Q00612	*Glucose-6-phosphate 1-dehydrogenase X*	20	9	59263	1,43E+06	3,95E+06	4,50E+06
P06745	*Glucose-6-phosphate isomerase*	23	12	62,767	1,79E+07	1,66E+07	1,31E+07
O08795	*Glucosidase 2 subunit beta*	8	4	58793	3,15E+06	2,00E+06	1,89E+06
P26443	*Glutamate dehydrogenase 1, mitochondrial*	20	10	61,337	1,30E+07	8,37E+06	1,04E+07
Q9D172	*Glutamine amidotransferase-like class 1 domain-containing protein 3*	20	4	28090	7,66E+06	1,01E+07	4,03E+06
P15105	*Glutamine synthetase*	26	9	42120	7,43E+06	1,63E+07	1,32E+07
A0A0A6YY34	*Glutathione peroxidase*	32	4	16354	9,59E+05	1,44E+06	1,31E+06
A0A0A6YVV2	*Glutathione peroxidase*	32	4	16483	9,59E+05	1,44E+06	1,31E+06
P11352	*Glutathione peroxidase 1*	23	4	22329	9,59E+05	1,44E+06	1,31E+06
Q6P8Q0	*Glutathione S-transferase*	27	6	25582	6,56E+07	1,87E+08	8,79E+07
Q9CPU4	*Glutathione S-transferase 3, mitochondrial*	18	2	16958	5,32E+05	4,57E+05	1,27E+05
P13745	*Glutathione S-transferase A1*	27	6	25608	6,56E+07	1,87E+08	8,79E+07

P24472	*Glutathione S-transferase A4*	42	8	25564	5,24E+07	1,10E+08	8,00E+07
Q9DCM2	*Glutathione S-transferase kappa 1*	7	1	25704	9,03E+05	1,15E+06	6,40E+05
P10649	*Glutathione S-transferase Mu 1*	56	9	25970	2,84E+07	3,75E+07	3,63E+07
P15626	*Glutathione S-transferase Mu 2*	58	6	25,717	7,21E+06	5,68E+06	7,31E+06
Q8R5I6	*Glutathione S-transferase Mu 4*	24	1	25,519		3,12E+05	2,62E+05
O09131	*Glutathione S-transferase omega-1*	41	14	27,498	5,45E+07	1,08E+08	9,49E+07
P19157	*Glutathione S-transferase P 1*	30	4	23609	1,49E+07	1,44E+07	1,88E+07
A2AE89	*Glutathione transferase*	50	9	28556	2,84E+07	3,75E+07	3,63E+07
P16858	*Glyceraldehyde-3-phosphate dehydrogenase*	55	13	35,81	1,11E+08	9,91E+07	1,01E+08
A0A0A0MQF6	*Glyceraldehyde-3-phosphate dehydrogenase*	51	13	38,653	1,11E+08	9,91E+07	1,01E+08
Q64521	*Glycerol-3-ph*	12	9	80954	2,26E+06	6,63E+06	7,14E+06
Q3ULJ0	*Glycerol-3-phosphate dehydrogenase 1-like protein*	5	1	38226		8,98E+05	2,15E+05
Q9D964	*Glycine amidinotransferase mitochondrial*	8	3	48297	7,46E+06	#DIV/0!	4,91E+06
Q9CZD3	*Glycine--tRNA ligase*	9	5	81878	1,03E+06	5,63E+05	2,17E+06
Q8CI94	*Glycogen phosphorylase, brain form*	20	11	96,73	5,16E+06	3,68E+06	3,62E+06
Q9WUB3	*Glycogen phosphorylase, muscle form*	15	6	97286	4,63E+06		
Q9CPV4	*Glyoxalase domain-containing protein 4*	14	3	33317	9,85E+05	9,01E+05	5,99E+05
E9Q197	*Glyoxalase domain-containing protein 4*	15	3	31207	9,85E+05	9,01E+05	5,99E+05
F6ZTG3	*Glyoxalase domain-containing protein 4 (Fragment)*	19	3	23700	9,85E+05	9,01E+05	5,99E+05
Q9WTP7	*GTP:AMP phosphotransferase AK3, mitochondrial*	26	5	25426	1,69E+06	1,55E+06	2,05E+06
P62827	*GTP-binding nuclear protein Ran*	31	6	24423	1,34E+07	1,67E+07	1,14E+07
Q9CQC9	*GTP-binding protein SAR1b*	11	2	22382	3,67E+06	3,87E+05	7,13E+05
P08752	*Guanine nucleotide-binding protein G(i) subunit alpha-2*	11	2	40489	5,04E+05	4,18E+05	3,63E+05
Q9DAS9	*Guanine nucleotide-binding protein G(I)/G(S)/G(O) subunit gamma-12*	57	4	7997	3,60E+06	1,35E+06	2,57E+06
D3YZX3	*Guanine nucleotide-binding protein G(I)/G(S)/G(T) subunit beta-2*	17	5	32408	5,34E+06	6,62E+06	4,78E+06
P62880	*Guanine nucleotide-binding protein G(I)/G(S)/G(T) subunit beta-2*	15	5	37331	5,34E+06	6,62E+06	4,78E+06
E9QKR0	*Guanine nucleotide-binding protein G(I)/G(S)/G(T) subunit beta-2*	13	5	41409	5,34E+06	6,62E+06	4,78E+06

Q6R0H7	*Guanine nucleotide-binding protein G(s) subunit alpha isoforms XLas*	3	2	121505	9,38E+05	1,62E+05	6,83E+05
Q9D034	*Guanine nucleotide-binding protein subunit alpha-13*	12	1	19937	#DIV/0!	4,45E+05	1,50E+05
P27601	*Guanine nucleotide-binding protein subunit alpha-13*	6	1	44055	#DIV/0!	4,45E+05	1,50E+05
A0A0N4SW28	*Guanine nucleotide-binding protein subunit gamma*	49	4	9182	3,60E+06	1,35E+06	2,57E+06
A0A0N4SVT3	*Guanine nucleotide-binding protein subunit gamma (Fragment)*	63	4	7197	3,60E+06	1,35E+06	2,57E+06
Q61696	*Heat shock 70 kDa protein 1A*	26	8	70079	1,39E+07	8,63E+06	6,18E+06
P17879	*Heat shock 70 kDa protein 1B*	26	8	70176	1,39E+07	8,63E+06	6,18E+06
Q61316	*Heat shock 70 kDa protein 4*	8	4	94133	2,75E+06	3,29E+06	2,65E+06
Q3U2G2	*Heat shock 70 kDa protein 4*	8	4	94209	2,75E+06	3,29E+06	2,65E+06
P63017	*Heat shock cognate 71 kDa protein*	46	16	70,871	8,48E+07	7,76E+07	7,06E+07
E9Q0U7	*Heat shock protein 105 kDa*	7	4	91678	7,77E+05	1,27E+06	1,01E+06
Q61699	*Heat shock protein 105 kDa*	7	4	96407	7,77E+05	1,27E+06	1,01E+06
Q9CQN1	*Heat shock protein 75 kDa mitochondrial*	3	1	80209			1,24E+06
P14602	*Heat shock protein beta-1*	58	10	23014	6,23E+07	1,87E+07	4,43E+07
P07901	*Heat shock protein HSP 90-alpha*	38	15	84,788	1,52E+07	1,74E+07	1,21E+07
P11499	*Heat shock protein HSP 90-beta*	40	16	83,281	9,55E+07	6,93E+07	8,47E+07
P17156	*Heat shock-related 70 kDa protein 2*	24	2	69642	1,27E+06		
A0A571BDN7	*HECT-type E3 ubiquitin transferase*	2	2	127651	7,53E+05	3,48E+05	4,95E+05
P01942	*Hemoglobin subunit alpha*	56	2	15085	4,96E+07	1,51E+07	2,78E+07
P02088	*Hemoglobin subunit beta-1*	95	2	15,84	9,88E+07	1,06E+08	7,55E+07
P02089	*Hemoglobin subunit beta-2*	95	7	15,878	6,61E+07	6,02E+07	6,18E+07
Q91X72	*Hemopexin*	14	5	51318	5,01E+06	5,57E+06	7,69E+06
P51859	*Hepatoma-derived growth factor*	11	2	26269	9,11E+05	9,83E+05	6,15E+05
E0CYW7	*Hepatoma-derived growth factor*	30	2	9412	9,11E+05	9,83E+05	6,15E+05
E0CXA0	*Hepatoma-derived growth factor (Fragment)*	12	2	22115	9,11E+05	9,83E+05	6,15E+05
Q99020	*Heterogeneous nuclear ribonucleoprotein A/B*	15	2	30831	5,94E+06	4,99E+05	6,08E+06
Q80XR6	*Heterogeneous nuclear ribonucleoprotein A/B*	14	2	33816	5,94E+06	4,99E+05	6,08E+06

Q20BD0	*Heterogeneous nuclear ribonucleoprotein A/B*	13	2	36211	5,94E+06	4,99E+05	6,08E+06
P49312	*Heterogeneous nuclear ribonucleoprotein A1*	21	4	34196	1,12E+06	1,71E+06	2,30E+06
Q5EBP8	*Heterogeneous nuclear ribonucleoprotein A1*	18	4	38834	1,12E+06	1,71E+06	2,30E+06
A2AL12	*Heterogeneous nuclear ribonucleoprotein A3*	29	6	34476	2,03E+07	1,46E+07	1,62E+07
Q8BG05	*Heterogeneous nuclear ribonucleoprotein A3*	24	6	39652	2,03E+07	1,46E+07	1,62E+07
G5E8G0	*Heterogeneous nuclear ribonucleoprotein D0*	16	4	30608	3,38E+06	2,70E+06	3,61E+06
G3X9W0	*Heterogeneous nuclear ribonucleoprotein D0*	14	4	36208	3,38E+06	2,70E+06	3,61E+06
Q60668	*Heterogeneous nuclear ribonucleoprotein D0*	13	4	38354	3,38E+06	2,70E+06	3,61E+06
F6ZV59	*Heterogeneous nuclear ribonucleoprotein D0 (Fragment)*	22	4	24730	3,38E+06	2,70E+06	3,61E+06
Q9Z2X1	*Heterogeneous nuclear ribonucleoprotein F*	18	4	45730	1,38E+06	3,22E+06	2,13E+06
O35737	*Heterogeneous nuclear ribonucleoprotein H*	15	3	49199	2,52E+06	3,68E+06	3,50E+06
Q8C2Q7	*Heterogeneous nuclear ribonucleoprotein H*	14	3	51218	2,52E+06	3,68E+06	3,50E+06
B2M1R6	*Heterogeneous nuclear ribonucleoprotein K*	24	8	48,562	8,80E+06	1,07E+07	8,93E+06
P61979	*Heterogeneous nuclear ribonucleoprotein K*	23	8	50,976	8,80E+06	1,07E+07	8,93E+06
Q9D0E1	*Heterogeneous nuclear ribonucleoprotein M*	13	9	77649	3,90E+06	2,45E+06	5,31E+06
G3UZI2	*Heterogeneous nuclear ribonucleoprotein Q*	15	4	58752	2,49E+06	2,89E+06	2,73E+06
F7B5B5	*Heterogeneous nuclear ribonucleoprotein R*	10	2	59626	9,98E+05	3,02E+05	3,95E+05
Q8VHM5	*Heterogeneous nuclear ribonucleoprotein R*	9	2	70888	9,98E+05	3,02E+05	3,95E+05
Q8VEK3	*Heterogeneous nuclear ribonucleoprotein U*	13	9	87918	1,18E+07	8,44E+06	1,07E+07
O88569	*Heterogeneous nuclear ribonucleoproteins A2/B1*	34	9	37403	3,70E+07	3,85E+07	3,72E+07
Q9Z204	*Heterogeneous nuclear ribonucleoproteins C1/C2*	16	5	34385	1,86E+06	2,33E+06	2,27E+06
P17710	*Hexokinase-1*	13	13	108303	5,28E+06	8,97E+06	7,04E+06
A0A0J9YUD8	*High mobility group box 1*	44	6	19,774	6,17E+06	6,21E+06	3,41E+06
A0A0J9YUZ4	*High mobility group box 1*	36	6	24,234	6,17E+06	6,21E+06	3,41E+06
P63158	*High mobility group protein B1*	35	6	24,894	6,17E+06	6,21E+06	3,41E+06
P10922	*Histone H1.0*	27	5	20861	1,28E+07	9,62E+06	1,12E+07
P43275	*Histone H1.1*	27	4	21785	3,29E+06	5,00E+06	1,45E+05

P15864	*Histone H1.2*	27	2	21267	6,80E+06	4,28E+06	5,46E+06
P43277	*Histone H1.3*	26	1	22100	5,57E+06		1,31E+06
P43274	*Histone H1.4*	26	3	21977	4,29E+07	3,35E+07	3,54E+07
P43276	*Histone H1.5*	25	5	22576	2,15E+07	2,07E+07	1,72E+07
A0A0N4SV66	*Histone H2A*	29	2	13660	3,37E+08	2,00E+08	2,86E+08
Q8CGP4	*Histone H2A*	28	2	14056	3,37E+08	2,00E+08	2,86E+08
C0HKE1	*Histone H2A type 1-B*	28	2	14135	3,37E+08	2,00E+08	2,86E+08
C0HKE2	*Histone H2A type 1-C*	28	2	14135	3,37E+08	2,00E+08	2,86E+08
C0HKE3	*Histone H2A type 1-D*	28	2	14135	3,37E+08	2,00E+08	2,86E+08
C0HKE4	*Histone H2A type 1-E*	28	2	14135	3,37E+08	2,00E+08	2,86E+08
Q8CGP5	*Histone H2A type 1-F*	28	2	14162	3,37E+08	2,00E+08	2,86E+08
C0HKE5	*Histone H2A type 1-G*	28	2	14135	3,37E+08	2,00E+08	2,86E+08
Q8CGP6	*Histone H2A type 1-H*	28	2	13950	3,37E+08	2,00E+08	2,86E+08
C0HKE6	*Histone H2A type 1-I*	28	2	14135	3,37E+08	2,00E+08	2,86E+08
Q8CGP7	*Histone H2A type 1-K*	28	2	14150	3,37E+08	2,00E+08	2,86E+08
C0HKE7	*Histone H2A type 1-N*	28	2	14135	3,37E+08	2,00E+08	2,86E+08
C0HKE8	*Histone H2A type 1-O*	28	2	14135	3,37E+08	2,00E+08	2,86E+08
C0HKE9	*Histone H2A type 1-P*	28	2	14135	3,37E+08	2,00E+08	2,86E+08
Q6GSS7	*Histone H2A type 2-A*	28	2	14095	3,37E+08	2,00E+08	2,86E+08
Q64523	*Histone H2A type 2-C*	28	2	13988	3,37E+08	2,00E+08	2,86E+08
Q8BFU2	*Histone H2A type 3*	28	2	14121	3,37E+08	2,00E+08	2,86E+08
Q8R1M2	*Histone H2A.J*	28	2	14045	3,37E+08	2,00E+08	2,86E+08
Q3THW5	*Histone H2A.V*	20	1	13509	9,96E+06	1,03E+07	7,77E+06
P0C0S6	*Histone H2A.Z*	20	1	13553	9,96E+06	1,03E+07	7,77E+06
P27661	*Histone H2AX*	27	1	15143	1,98E+06		1,01E+06
Q8CBB6	*Histone H2B*	52	1	14888	2,55E+05		
Q64475	*Histone H2B type 1-B*	52	1	13952	1,85E+06		

Q6ZWY9	*Histone H2B type 1-C/E/G*	56	1	13906	2,55E+05		
P10853	*Histone H2B type 1-F/J/L*	56	1	13936	2,55E+05		
Q64478	*Histone H2B type 1-H*	56	1	13920	2,55E+05		
Q8CGP1	*Histone H2B type 1-K*	56	1	13920	2,55E+05		
F8WI35	*Histone H3*	31	7	15199	5,47E+08	5,25E+08	5,24E+08
P68433	*Histone H3.1*	31	7	15404	5,47E+08	5,25E+08	5,24E+08
A0A1W2P768	*Histone H3.2*	23	7	20247	5,47E+08	5,25E+08	5,24E+08
P84228	*Histone H3.2*	31	7	15388	5,47E+08	5,25E+08	5,24E+08
P84244	*Histone H3.3*	31	7	15328	5,47E+08	5,25E+08	5,24E+08
P02301	*Histone H3.3C*	31	7	15315	5,47E+08	5,25E+08	5,24E+08
P62806	*Histone H4*	52	7	11367	1,05E+09	9,82E+08	9,92E+08
Q8VHD8	*Hornerin*	5	7	247585	1,09E+07	2,08E+05	1,16E+07
E9QNP3	*Hornerin*	3	7	323369	1,09E+07	2,08E+05	1,16E+07
F8WJ23	*Hornerin*	3	7	340666	1,09E+07	2,08E+05	1,16E+07
Q61425	*Hydroxyacyl-coenzyme A dehydrogenase mitochondrial*	17	6	34464	2,08E+07	1,82E+07	1,73E+07
A2AFQ2	*Hydroxysteroid (17-beta) dehydrogenase 10*	26	4	28,374	7,57E+05	4,61E+05	4,62E+05
Q9JKR6	*Hypoxia up-regulated protein 1*	24	19	111,181	2,26E+07	6,86E+06	1,68E+07
F6TQW2	*Immunoglobulin heavy constant gamma 2C (Fragment)*	12	2	44214	1,06E+07	1,00E+07	6,98E+06
A0A0A6YY53	*Immunoglobulin heavy constant gamma 2C (Fragment)*	15	2	36527	1,06E+07	1,00E+07	6,98E+06
P01837	*Immunoglobulin kappa constant*	43	2	11934	1,06E+06	5,58E+05	1,11E+06
P70168	*Importin subunit beta-1*	7	4	97184	9,00E+05	3,48E+05	1,07E+06
Q5BKQ4	*Inactive pancreatic lipase-related protein 1*	32	11	52696	3,14E+07		4,00E+07
Q9D819	*Inorganic pyroph*	17	5	32667	5,41E+06	1,38E+06	3,38E+06
Q924B0	*Inositol-1-monophosphatase*	28	6	30429	4,46E+06	4,04E+06	1,83E+06
Q9JHR7	*Insulin-degrading enzyme*	3	2	117772	7,95E+05		5,66E+05
Q8CGB9	*Insulin-degrading enzyme*	3	2	117695	7,95E+05		5,66E+05
Q3V3R4	*Integrin alpha-1*	7	7	130810	2,10E+06	1,92E+06	2,71E+06

P11688	*Integrin alpha-5*	3	3	115043	8,88E+05	3,59E+05	6,65E+05
P09055	*Integrin beta-1*	12	9	88231	1,61E+07	8,21E+06	8,81E+06
A0A2K6EDJ7	*Inter alpha-trypsin inhibitor heavy chain 4*	2	1	100324	3,00E+05	5,38E+05	4,87E+05
E9Q5L2	*Inter alpha-trypsin inhibitor heavy chain 4*	2	1	102823	3,00E+05	5,38E+05	4,87E+05
E9PVD2	*Inter alpha-trypsin inhibitor heavy chain 4*	2	1	104588	3,00E+05	5,38E+05	4,87E+05
A6X935	*Inter alpha-trypsin inhibitor heavy chain 4*	2	1	104660	3,00E+05	5,38E+05	4,87E+05
D3YTT4	*Isobutyryl-CoA dehydrogenase mitochondrial*	8	3	45076	6,11E+05	3,49E+06	2,47E+06
A0A0R4J0P1	*Isobutyryl-CoA dehydrogenase mitochondrial*	8	3	45092	6,11E+05	3,49E+06	2,47E+06
Q9D7B6	*Isobutyryl-CoA dehydrogenase mitochondrial*	8	3	45020	6,11E+05	3,49E+06	2,47E+06
A0A1L1STE6	*Isocitrate dehydrogenase [NAD] subunit mitochondrial*	19	7	41520	1,40E+07	1,22E+07	1,25E+07
Q91VA7	*Isocitrate dehydrogenase [NAD] subunit mitochondrial*	12	5	42195	2,78E+06	9,21E+05	3,63E+06
A0A668KL51	*Isocitrate dehydrogenase [NAD] subunit mitochondrial*	13	5	41700	2,78E+06	9,21E+05	3,63E+06
Q9D6R2	*Isocitrate dehydrogenase [NAD] subunit alpha mitochondrial*	20	7	39639	1,40E+07	1,22E+07	1,25E+07
O88844	*Isocitrate dehydrogenase [NADP] cytoplasmic*	23	9	46674	1,36E+07	6,99E+06	5,42E+06
P54071	*Isocitrate dehydrogenase [NADP], mitochondrial*	38	16	50,906	3,95E+07	3,50E+07	3,08E+07
Q9JHI5	*Isovaleryl-CoA dehydrogenase mitochondrial*	8	3	46325	7,06E+05	2,97E+06	3,33E+06
Q02257	*Junction plakoglobin*	12	8	81,801	2,55E+06	5,04E+05	2,86E+06
P15947	*Kallikrein-1*	18	4	28775	8,96E+05		5,98E+06
Q497I4	*Keratin type I cuticular Ha5*	7	1	50530			8,68E+06
P19001	*Keratin type I cyt*	69	23	44542	2,08E+08	4,53E+08	2,51E+08
P05784	*Keratin type I cyt*	32	12	47538	4,92E+07	7,77E+07	5,48E+07
Q61414	*Keratin type I cyt*	36	8	49138	2,66E+07	7,61E+05	7,22E+06
B1AQ77	*Keratin type I cyt*	36	8	49494	2,66E+07	7,61E+05	7,22E+06
Q9QWL7	*Keratin type I cyt*	30	3	48162	8,03E+05		5,02E+05
Q6IFX2	*Keratin type I cyt*	18	2	50133	1,68E+07	3,13E+07	1,77E+07
Q9Z2K1	*Keratin type I cyt*	15	1	51606	2,16E+06	2,99E+06	
Q9D312	*Keratin type I cyt*	15	6	49034	1,59E+06	3,09E+06	1,64E+06

P11679	*Keratin type II cyt*	65	26	54565	3,51E+08	6,29E+08	3,29E+08
Q9DCV7	*Keratin type II cyt*	25	7	50709	2,45E+06	3,79E+06	1,29E+06
Q6IFZ6	*Keratin type II cyt*	8	1	61359	1,45E+07	1,31E+07	9,94E+06
Q8VED5	*Keratin type II cyt*	9	1	57552	2,00E+06	6,76E+05	1,39E+06
Q6IME9	*Keratin type II cyt*	8	1	56750	1,50E+07		1,87E+07
A2A513	*Keratin 10*	47	22	57,041	4,94E+08	6,52E+07	7,73E+08
E9Q0F0	*Keratin 78*	25	24	112,265	3,77E+07	2,18E+06	3,03E+07
Q61765	*Keratin, type I cuticular Ha1*	17	1	47,117			3,66E+05
Q8K0Y2	*Keratin, type I cuticular Ha3-I*	18	1	46,137	1,15E+07		1,14E+07
P02535	*Keratin, type I cytoskeletal 10*	46	22	57,77	4,94E+08	6,52E+07	7,73E+08
P08730	*Keratin, type I cytoskeletal 13*	56	16	47,754	3,66E+08	2,90E+07	3,21E+08
Q61781	*Keratin, type I cytoskeletal 14*	44	7	52,867	2,77E+07		1,15E+07
Q9ERE2	*Keratin, type II cuticular Hb1*	22	2	52,863	4,53E+06		7,03E+06
Q9Z2T6	*Keratin, type II cuticular Hb5*	23	4	55,759			1,88E+07
P97861	*Keratin, type II cuticular Hb6*	21	2	53,251	4,53E+06		7,03E+06
P04104	*Keratin, type II cytoskeletal 1*	60	30	65,606	3,67E+08	4,65E+07	8,00E+08
Q3TTY5	*Keratin, type II cytoskeletal 2 epidermal*	7	1	70,923	2,10E+06	1,37E+06	
Q3UV17	*Keratin, type II cytoskeletal 2 oral*	8	1	62,845	1,19E+07	3,98E+06	2,08E+07
P07744	*Keratin, type II cytoskeletal 4*	52	31	56,283	5,36E+08	2,95E+07	5,49E+08
Q922U2	*Keratin, type II cytoskeletal 5*	46	20	61,767	8,77E+07	7,48E+06	6,32E+07
P50446	*Keratin, type II cytoskeletal 6A*	29	2	59,335	3,36E+06		3,60E+06
Q9Z331	*Keratin, type II cytoskeletal 6B*	26	2	60,322	3,36E+06		2,22E+06
Q61029	*Lamina-associated polypeptide 2 isoforms beta/delta/epsilon/gamma*	8	1	50373	7,41E+05		3,37E+05
P14733	*Lamin-B1*	12	6	66786	6,19E+06	6,26E+06	6,85E+06
P97927	*Laminin subunit alpha-4*	3	3	201818	9,70E+05	4,16E+05	2,28E+05
Q61001	*Laminin subunit alpha-5*	2	6	404056	2,78E+06		2,99E+06
P02469	*Laminin subunit beta-1*	6	10	197089	1,38E+06	2,49E+05	5,67E+05

E9QN70	*Laminin subunit beta-1*	6	10	202440	1,38E+06	2,49E+05	5,67E+05
Q61292	*Laminin subunit beta-2*	7	11	196578	6,04E+06	1,84E+06	2,13E+06
F8VQJ3	*Laminin subunit gamma-1*	12	16	177187	1,99E+07	1,02E+07	1,32E+07
P47963	*Large ribosomal subunit protein eL13*	42	9	24305	5,23E+07	3,40E+07	3,65E+07
Q9CR57	*Large ribosomal subunit protein eL14*	29	6	23564	2,24E+07	1,47E+07	2,18E+07
Q9CZM2	*Large ribosomal subunit protein eL15*	36	7	24146	2,24E+07	1,36E+07	1,82E+07
P35980	*Large ribosomal subunit protein eL18*	33	6	21645	4,16E+07	1,83E+07	3,59E+07
P84099	*Large ribosomal subunit protein eL19*	16	2	23466	1,68E+07	1,00E+07	1,57E+07
P62717	*Large ribosomal subunit protein eL20*	40	7	20732	1,31E+07	3,59E+06	7,60E+06
P67984	*Large ribosomal subunit protein eL22*	23	3	14759	1,12E+07	9,94E+06	1,07E+07
Q8BP67	*Large ribosomal subunit protein eL24*	30	5	17,779	8,07E+06	5,67E+06	8,32E+06
P61358	*Large ribosomal subunit protein eL27*	39	5	15798	1,39E+07	5,86E+06	1,52E+07
P41105	*Large ribosomal subunit protein eL28*	38	6	15733	2,10E+07	1,25E+07	2,20E+07
P47915	*Large ribosomal subunit protein eL29*	19	3	17587	6,53E+06	5,88E+06	5,99E+06
P62889	*Large ribosomal subunit protein eL30*	58	5	12,784	1,49E+07	3,61E+06	7,10E+06
P62900	*Large ribosomal subunit protein eL31*	25	3	14463	1,86E+07	1,27E+07	2,12E+07
P62911	*Large ribosomal subunit protein eL32*	10	1	15860	1,28E+07	3,26E+06	3,61E+06
O55142	*Large ribosomal subunit protein eL33*	32	4	12554	1,10E+07	3,05E+06	7,72E+06
Q9D1R9	*Large ribosomal subunit protein eL34*	28	4	13293	1,80E+07	1,29E+07	1,78E+07
Q6ZWZ4	*Large ribosomal subunit protein eL36*	20	2	12,254	7,20E+06	2,88E+06	6,28E+06
P83882	*Large ribosomal subunit protein eL42*	32	5	12441	1,67E+07	6,02E+06	7,29E+06
P61514	*Large ribosomal subunit protein eL43*	45	4	10275	4,78E+06	7,40E+05	1,26E+06
P47911	*Large ribosomal subunit protein eL6*	41	13	33510	5,24E+07	3,37E+07	4,60E+07
P12970	*Large ribosomal subunit protein eL8*	42	14	29,977	4,92E+07	2,34E+07	4,64E+07
P47955	*Large ribosomal subunit protein P1*	19	1	11475	2,78E+07	6,72E+06	1,37E+07
P99027	*Large ribosomal subunit protein P2*	69	5	11651	2,60E+07	1,43E+07	2,05E+07
P35979	*Large ribosomal subunit protein uL11*	59	8	17805	2,57E+07	8,56E+06	1,67E+07

P19253	*Large ribosomal subunit protein uL13*	36	9	23464	2,91E+07	3,00E+06	1,56E+07
P62830	*Large ribosomal subunit protein uL14*	33	4	14865	1,54E+07	9,36E+06	1,47E+07
P14115	*Large ribosomal subunit protein uL15*	28	4	16605	3,12E+07	1,58E+07	2,82E+07
Q6ZWV3	*Large ribosomal subunit protein uL16*	40	7	24,604	1,57E+07	5,01E+06	5,61E+06
P62918	*Large ribosomal subunit protein uL2*	19	5	28025	3,30E+07	2,37E+07	3,37E+07
Q6ZWZ7	*Large ribosomal subunit protein uL22*	27	4	21397	1,55E+07	1,18E+07	1,82E+07
Q9CPR4	*Large ribosomal subunit protein uL22*	27	4	21423	1,55E+07	1,18E+07	1,82E+07
P62751	*Large ribosomal subunit protein uL23*	26	5	17695	1,71E+07	7,71E+06	1,23E+07
P61255	*Large ribosomal subunit protein uL24*	29	6	17258	2,42E+07	8,60E+06	2,30E+07
Q6ZWV7	*Large ribosomal subunit protein uL29*	26	3	14553	6,30E+06	4,22E+06	3,50E+06
P27659	*Large ribosomal subunit protein uL3*	29	11	46,11	4,26E+07	1,48E+07	2,08E+07
P14148	*Large ribosomal subunit protein uL30*	39	13	31420	4,73E+07	2,64E+07	4,47E+07
Q9D8E6	*Large ribosomal subunit protein uL4*	35	14	47,154	2,70E+07	1,60E+07	3,19E+07
Q9CXW4	*Large ribosomal subunit protein uL5*	17	3	20252	2,36E+07	3,30E+06	1,01E+07
A0A0G2JES3	*Large ribosomal subunit protein uL6*	15	3	21741	6,47E+06	2,86E+06	4,01E+06
P51410	*Large ribosomal subunit protein uL6*	15	3	21881	6,47E+06	2,86E+06	4,01E+06
P47962	*Larger ribosomal subunit protein uL18*	27	9	34401	2,57E+07	4,72E+06	1,61E+07
F8VPR1	*Leiomodin-1*	4	2	66268	3,31E+06	1,46E+06	2,48E+06
Q8BVA4	*Leiomodin-1*	4	2	66310	3,31E+06	1,46E+06	2,48E+06
Q922Q8	*Leucine-rich repeat-containing protein 59*	41	9	34877	1,79E+07	6,18E+06	1,22E+07
Q8BMJ2	*Leucine--tRNA ligase cytoplasmic*	7	6	134192	2,46E+06		4,74E+05
Q9D154	*Leukocyte elastase inhibitor A*	29	9	42575	1,36E+07	2,15E+06	6,12E+06
Q61792	*LIM and SH3 domain protein 1*	27	8	29994	3,08E+06	6,00E+06	3,03E+06
A2A6H0	*LIM and SH3 domain protein 1 (Fragment)*	38	8	21288	3,08E+06	6,00E+06	3,03E+06
Q9ERG0	*LIM domain and actin-binding protein 1*	4	3	84060	#DIV/0!	1,64E+05	5,56E+05
P53395	*Lipoamide acyltransferase component*	19	9	53247	3,14E+06	5,44E+06	6,58E+06
Q8BFW7	*Lipoma-preferred partner homolog*	23	10	65,891	1,62E+07	6,04E+06	5,85E+06

A0A1B0GSX0	*L-lactate dehydrogenase*	28	9	39,758	3,19E+07	2,18E+07	2,83E+07
A0A6I8MX27	*L-lactate dehydrogenase*	36	10	37,349	2,35E+07	3,47E+07	2,48E+07
P06151	*L-lactate dehydrogenase A chain*	30	9	36,499	3,19E+07	2,18E+07	2,83E+07
P16125	*L-lactate dehydrogenase B chain*	37	10	36,572	2,35E+07	3,47E+07	2,48E+07
Q8CGK3	*Lon protease homolog mitochondrial*	4	4	105843	3,38E+06	1,19E+06	1,62E+06
A0A0R4J083	*Long-chain specific acyl-CoA dehydrogenase mitochondrial*	18	8	47897	8,85E+06	1,43E+07	9,03E+06
D3Z041	*Long-chain-fatty-acid--CoA ligase*	6	2	78035	1,62E+06	2,47E+06	2,48E+06
P41216	*Long-chain-fatty-acid--CoA ligase 1*	6	2	77952	1,62E+06	2,47E+06	2,48E+06
Q8JZR0	*Long-chain-fatty-acid--CoA ligase 5*	4	1	76206	1,19E+06		
P18165	*Loricrin*	5	2	38200	4,70E+06		1,79E+07
P51885	*Lumican*	32	10	38265	3,57E+07	1,98E+07	2,34E+07
A6PWS5	*Macrophage-capping protein (Fragment)*	18	1	28060	8,44E+05		
Q9EQK5	*Major vault protein*	7	4	95924	3,46E+06	3,28E+06	1,59E+06
E9Q3X0	*Major vault protein*	7	4	96857	3,46E+06	3,28E+06	1,59E+06
A0A5F8MPN8	*Malate dehydrogenase*	28	11	39005	5,29E+07	5,29E+07	6,50E+07
P14152	*Malate dehydrogenase cytoplasmic*	30	11	36511	5,29E+07	5,29E+07	6,50E+07
P08249	*Malate dehydrogenase, mitochondrial*	68	19	35,611	2,21E+08	1,76E+08	1,37E+08
Q6ZQI3	*Malectin*	10	3	32342	1,80E+06	8,36E+05	1,59E+06
Q3TQP6	*Malic enzyme*	11	5	61481	1,67E+06	3,22E+06	2,67E+06
Q80UM7	*Mannosyl-oligosaccharide glucosidase*	4	2	91831	1,96E+06	4,73E+05	8,49E+05
P45952	*Medium-chain specific acyl-CoA dehydrogenase mitochondrial*	26	11	46481	8,15E+06	1,30E+07	8,54E+06
O70423	*Membrane primary amine oxidase*	14	9	84534	6,73E+06	3,14E+06	1,36E+06
Q3TMX5	*Mesencephalic astrocyte-derived neurotrophic factor*	17	4	20388	2,23E+07	2,64E+06	3,92E+06
Q9CXI5	*Mesencephalic astrocyte-derived neurotrophic factor*	17	4	20374	2,23E+07	2,64E+06	3,92E+06
F6USD5	*Mesencephalic astrocyte-derived neurotrophic factor (Fragment)*	26	4	13756	2,23E+07	2,64E+06	3,92E+06
F6T4L3	*Mesencephalic astrocyte-derived neurotrophic factor (Fragment)*	24	4	14482	2,23E+07	2,64E+06	3,92E+06
P17563	*Methanethiol oxidase*	8	4	52514	2,17E+06	3,71E+06	3,08E+06

Q3ULD5	*Methylcrotonoyl-CoA carboxylase beta chain mitochondrial*	9	5	61379	3,31E+06	2,85E+06	1,83E+06
Q99MR8	*Methylcrotonoyl-CoA carboxylase subunit alpha mitochondrial*	6	4	79344	2,28E+06	4,50E+06	2,68E+06
Q9EQ20	*Methylmalonate-semialdehyde/malonate-semialdehyde dehydrogenase*	32	15	57,916	3,18E+07	4,68E+07	3,97E+07
Q8C6B0	*Methyltransferase-like 7A1*	10	1	28096			8,60E+05
Q9CRB9	*MICOS complex subunit MIC16*	27	6	26335	6,47E+06	5,90E+06	4,40E+06
Q8CAQ8	*MICOS complex subunit Mic60*	19	6	83900	2,76E+06	4,18E+06	3,67E+06
A0A0U1RP81	*MICOS complex subunit MIC60*	18	1	53717	8,14E+05	1,02E+06	8,42E+05
Q9D1H9	*Microfibril-associated glycoprotein 4*	14	4	28938	7,46E+06	5,40E+05	3,13E+06
O08601	*Microsomal triglyceride transfer protein large subunit*	16	12	99099	1,43E+07		
Q62000	*Mimecan*	17	5	34012	6,87E+06	7,04E+06	4,88E+06
Q8K009	*Mitochondrial 10-formyltetrahydrofolate dehydrogenase*	5	3	101590	3,65E+06		2,87E+06
Q9CR62	*Mitochondrial 2-oxoglutarate/malate carrier protein*	13	4	34155	3,37E+06	1,46E+06	4,12E+06
A2AFW6	*Mitochondrial carrier homolog 2*	11	3	32345	8,06E+05	7,82E+05	2,91E+06
Q791V5	*Mitochondrial carrier homolog 2*	11	3	33499	8,06E+05	7,82E+05	2,91E+06
Q9D050	*Mitochondrial carrier homolog 2*	10	3	34554	8,06E+05	7,82E+05	2,91E+06
P26041	*Moesin*	19	6	67767	7,10E+06	6,89E+06	3,14E+06
E9QAQ8	*Mucin 5, subtypes A and C, tracheobronchial/gastric*	10	28	372,17	1,06E+07	5,33E+07	4,12E+07
O35682	*Myeloid-associated differentiation marker*	8	2	35285	6,54E+06	3,27E+06	1,23E+06
A0A0N4SW94	*Myeloid-associated differentiation marker (Fragment)*	33	2	8640	6,54E+06	3,27E+06	1,23E+06
P05977	*Myosin light chain 1/3, skeletal muscle isoform*	35	3	20594	1,95E+07		
P09542	*Myosin light chain 3*	57	7	22,422	2,86E+07	3,12E+06	
Q8CI43	*Myosin light chain 6B*	10	1	22749	1,09E+06	2,80E+06	3,76E+05
B1B1A8	*Myosin light chain kinase, smooth muscle*	14	26	213,609	1,26E+08	7,78E+07	7,41E+07
Q6PDN3	*Myosin light chain kinase, smooth muscle*	14	26	212,925	1,26E+08	7,78E+07	7,41E+07
A0A0U1RP93	*Myosin light chain, phosphorylatable, fast skeletal muscle*	58	9	16,881	6,16E+07		
Q60605	*Myosin light polypeptide 6*	56	6	16,93	2,06E+08	1,66E+08	1,47E+08
P97457	*Myosin regulatory light chain 11*	51	9	18,955	6,16E+07		

Q3THE2	*Myosin regulatory light chain 12B*	51	2	19779	9,96E+06	1,06E+07	1,00E+07
Q9CQ19	*Myosin regulatory light polypeptide 9*	62	3	19,854	9,04E+07	8,96E+07	9,19E+07
A0A338P6K2	*Myosin, h+H2:H1240eavy polypeptide 11, smooth muscle*	58	1	22787	1,63E+07	1,59E+07	1,15E+07
Q5SV64	*Myosin, heavy polypeptide 10, non-muscle*	10	2	232,472	1,14E+06	1,79E+06	5,03E+06
Q3UH59	*Myosin, heavy polypeptide 10, non-muscle*	10	2	233,449	1,14E+06	1,79E+06	5,03E+06
A0A2R8VHF9	*Myosin, heavy polypeptide 11, smooth muscle*	58	1	223357	5,83E+06	3,91E+06	2,83E+06
B1AR69	*Myosin, heavy polypeptide 13, skeletal muscle*	11	1	223,561	8,34E+06		
K3W4R2	*Myosin, heavy polypeptide 14*	7	4	228559		9,77E+05	1,07E+05
G3UW82	*Myosin, heavy polypeptide 2, skeletal muscle, adult*	26	1	223,219	3,36E+05		
E9PWG4	*Myosin, light polypeptide 1*	40	3	17689	1,95E+07		
A0A0G2JDW2	*Myosin, light polypeptide 3*	64	7	20,183	2,86E+07	3,12E+06	
A0A1W2P6F6	*Myosin, light polypeptide 6, alkali, smooth muscle and non-muscle*	55	6	17,005	2,06E+08	1,66E+08	1,47E+08
A0A1W2P7Q9	*Myosin, light polypeptide 6, alkali, smooth muscle and non-muscle*	53	6	17,673	2,06E+08	1,66E+08	1,47E+08
Q5SX40	*Myosin-1*	34	10	223,342	5,89E+07		
Q61879	*Myosin-10*	10	2	228,996	1,14E+06	1,79E+06	5,03E+06
O08638	*Myosin-11*	56	1	227028	4,00E+06	2,06E+06	4,43E+06
Q6URW6	*Myosin-14*	7	4	228584		9,77E+05	1,07E+05
A2AQP0	*Myosin-7B*	4	1	221495			
P13542	*Myosin-8*	34	13	222,708	2,64E+07		
Q8VDD5	*Myosin-9*	28	31	226,372	2,28E+07	2,76E+07	2,62E+07
P26645	*Myristoylated alanine-rich C-kinase substrate*	6	1	29661	5,63E+05	8,63E+05	6,82E+05
P70441	*Na(+)/H(+) exchange regulatory cofactor NHE-RF1*	8	3	38600	1,94E+06	1,09E+06	1,85E+06
Q61941	*NAD(P) transhydrogenase mitochondrial*	5	5	113838	3,00E+06	2,92E+06	3,48E+06
Q64669	*NAD(P)H dehydrogenase [quinone] 1*	30	8	30960	9,69E+06	3,35E+07	5,94E+07
Q99KE1	*NAD-dependent malic enzyme mitochondrial*	7	3	65799	1,50E+06	3,76E+06	1,88E+06
Q99LC3	*NADH dehydrogenase [ubiquinone] 1 alpha subcomplex subunit 10*	26	6	40603	4,11E+06	3,04E+06	5,44E+06
Q9D8B4	*NADH dehydrogenase [ubiquinone] 1 alpha subcomplex subunit 11*	24	2	14982	1,94E+06	2,06E+06	1,31E+06

G5E814	*NADH dehydrogenase [ubiquinone] 1 alpha subcomplex subunit 11*	24	2	15115	1,94E+06	2,06E+06	1,31E+06
Q9CPP6	*NADH dehydrogenase [ubiquinone] 1 alpha subcomplex subunit 5*	45	4	13360	1,87E+06	1,01E+06	1,37E+06
A0A0R3P9C8	*NADH dehydrogenase [ubiquinone] 1 alpha subcomplex subunit 9*	25	6	42122	3,14E+06	8,19E+05	3,23E+06
Q9DC69	*NADH dehydrogenase [ubiquinone] 1 alpha subcomplex subunit 9*	25	6	42525	3,14E+06	8,19E+05	3,23E+06
Q9DCS9	*NADH dehydrogenase [ubiquinone] 1 beta subcomplex subunit 10*	27	3	21024	1,57E+06	1,67E+06	2,28E+06
Q9CQC7	*NADH dehydrogenase [ubiquinone] 1 beta subcomplex subunit 4*	27	2	15081	3,54E+05	2,71E+06	1,32E+06
D3Z568	*NADH dehydrogenase [ubiquinone] 1 beta subcomplex subunit 5*	29	4	14038	1,29E+06	2,05E+06	1,09E+06
Q9CQH3	*NADH dehydrogenase [ubiquinone] 1 beta subcomplex subunit 5*	18	4	21710	1,29E+06	2,05E+06	1,09E+06
D3YUM1	*NADH dehydrogenase [ubiquinone] flavoprotein 1 mitochondrial*	24	8	49913	5,74E+06	3,33E+06	5,30E+06
Q91YT0	*NADH dehydrogenase [ubiquinone] flavoprotein 1 mitochondrial*	23	8	50834	5,74E+06	3,33E+06	5,30E+06
Q9D6J6	*NADH dehydrogenase [ubiquinone] flavoprotein 2 mitochondrial*	17	3	27285	2,42E+06	1,34E+06	1,75E+06
D3YXT0	*NADH dehydrogenase [ubiquinone] iron-sulfur protein 2*	5	2	49573	4,63E+06	3,70E+06	4,76E+06
Q91WD5	*NADH dehydrogenase [ubiquinone] iron-sulfur protein 2*	4	2	52626	4,63E+06	3,70E+06	4,76E+06
A0A0A6YXD3	*NADH dehydrogenase [ubiquinone] iron-sulfur protein 2*	14	2	16897	4,63E+06	3,70E+06	4,76E+06
A0A0A6YW30	*NADH dehydrogenase [ubiquinone] iron-sulfur protein 2*	9	2	26788	4,63E+06	3,70E+06	4,76E+06
Q9DCT2	*NADH dehydrogenase [ubiquinone] iron-sulfur protein 3*	21	5	30149	9,53E+06	8,23E+06	8,16E+06
F2Z456	*NADH-cytochrome b5 reductase*	13	4	34929	1,37E+07	9,67E+06	5,65E+06
Q9DCN2	*NADH-cytochrome b5 reductase 3*	14	4	34128	1,37E+07	9,67E+06	5,65E+06
Q91VD9	*NADH-ubiquinone oxidoreductase 75 kDa subunit mitochondrial*	18	10	79777	8,51E+06	1,23E+07	1,12E+07
P06801	*NADP-dependent malic enzyme*	10	5	63954	1,67E+06	3,22E+06	2,67E+06
P70670	*Nascent polypeptide-associated complex subunit alpha muscle-specific*	2	4	220497	6,80E+06	5,61E+06	4,97E+06
Q8BHN3	*Neutral alpha-gluc*	10	7	106911	1,81E+06	1,86E+06	1,41E+06
P10493	*Nidogen-1*	9	9	136538	5,93E+06	3,82E+06	4,15E+06
Q9CZ44	*NSFL1 cofactor p47*	12	4	40710	1,13E+06	1,62E+06	1,35E+06
A0A1C7CYU3	*Nucleobindin-1*	9	3	52997	4,24E+05	1,56E+05	2,76E+05
Q02819	*Nucleobindin-1*	8	3	53409	4,24E+05	1,56E+05	2,76E+05
Q3UKN6	*Nucleobindin-2*	10	3	50354	1,77E+06	1,28E+06	6,72E+05

P81117	*Nucleobindin-2*	10	3	50305	1,77E+06	1,28E+06	6,72E+05
P09405	*Nucleolin*	24	16	76723	1,81E+07	1,41E+07	1,89E+07
Q61937	*Nucleophosmin*	20	6	32560	1,52E+07	9,55E+06	5,88E+06
Q5SQB0	*Nucleophosmin*	22	6	29525	1,52E+07	9,55E+06	5,88E+06
E9PZF0	*Nucleoside diphosphate kinase*	43	4	30,2	2,77E+07	1,27E+07	1,83E+07
P15532	*Nucleoside diphosphate kinase A*	53	1	17,208	8,41E+05		
Q9CZ30	*Obg-like ATPase 1*	9	3	44730	6,21E+05	3,00E+05	4,31E+05
A0A0J9YU93	*OCIA domain-containing protein 2*	25	2	9875	1,20E+06	1,52E+06	1,20E+06
Q9D8W7	*OCIA domain-containing protein 2*	15	2	16926	1,20E+06	1,52E+06	1,20E+06
P29758	*Ornithine aminotransferase mitochondrial*	17	6	48355	1,93E+06	1,32E+06	2,02E+06
A0A494BA97	*Osteoclast stimulating factor 1*	17	2	23394	2,78E+05	2,32E+05	5,48E+05
Q62422	*Osteoclast-stimulating factor 1*	17	2	23783	2,78E+05	2,32E+05	5,48E+05
Z4YJV4	*Oxoglutarate dehydrogenase (succinyl-transferring)*	16	15	116,028	1,11E+07	1,82E+07	1,56E+07
P14206	*P14206*	44	10	32,838	3,71E+07	1,98E+07	3,19E+07
Q9ET54	*Palladin*	6	8	152131	1,10E+07	5,66E+06	1,54E+06
J3QP56	*Palmitoyl-protein hydrolase*	12	2	23726	5,63E+05	5,89E+05	4,21E+05
P00688	*Pancreatic alpha-amylase 2a5*	48	13	57,318	3,95E+08	1,08E+06	4,10E+08
P17892	*Pancreatic lipase-related protein 2*	9	2	54017	6,41E+06	#DIV/0!	8,26E+06
Q6P8U6	*Pancreatic triacylglycerol lipase*	39	12	51428	1,66E+08		1,66E+08
Q9D0J8	*Parathym*	23	3	11430	5,10E+06	9,49E+06	9,18E+06
Q99LX0	*Parkinson disease protein 7 homolog*	26	4	20021	2,51E+06	1,93E+06	4,18E+06
O70209	*PDZ and LIM domain protein 3*	29	6	34300	5,95E+06	6,77E+06	4,55E+06
Q3TJD7	*PDZ and LIM domain protein 7*	21	9	50,119	8,34E+06	7,56E+06	6,04E+06
P17742	*Peptidyl-prolyl cis-trans isomerase A*	55	7	17,971	8,71E+07	5,40E+07	7,95E+07
P24369	*Peptidyl-prolyl cis-trans isomerase B*	44	9	23713	6,67E+07	3,14E+07	6,19E+07
P30412	*Peptidyl-prolyl cis-trans isomerase C*	9	1	22794	7,99E+05	2,30E+05	2,97E+05
Q9D1M7	*Peptidyl-prolyl cis-trans isomerase FKBP11*	14	3	22137	1,61E+07		9,77E+06

Q62009	*Periostin*	19	11	93144	1,20E+07	7,58E+06	6,25E+06
G5E898	*Periplakin*	9	15	203778	2,09E+06		1,81E+06
P35700	*Peroxiredoxin-1*	61	9	22,176	1,02E+08	1,11E+08	7,33E+07
Q61171	*Peroxiredoxin-2*	42	9	21779	2,38E+07	2,69E+07	2,84E+07
O08807	*Peroxiredoxin-4*	25	4	31053	1,31E+07	5,70E+05	3,89E+06
P99029	*Peroxiredoxin-5 mitochondrial*	40	9	21897	3,44E+07	3,76E+07	3,70E+07
O08709	*Peroxiredoxin-6*	21	2	24871	4,56E+06	2,03E+06	3,85E+06
P70296	*Phosphatidylethanolamine-binding protein 1*	33	4	20830	8,70E+06	5,51E+06	8,27E+06
P53810	*Phosphatidylinositol transfer protein alpha isoform*	11	2	31893	8,78E+05	6,09E+05	9,22E+05
J3QQ30	*Phosphatidylinositol transfer protein, alpha*	11	2	31837	8,78E+05	6,09E+05	9,22E+05
J3QPW1	*Phosphatidylinositol transfer protein, alpha*	11	2	32022	8,78E+05	6,09E+05	9,22E+05
Q7M6Y3	*Phosphatidylinositol-binding clathrin assembly protein*	5	1	71543	3,02E+05		
A0A2I3BS39	*Phosphoenolpyruvate carboxykinase (GTP)*	8	4	66910	4,36E+06		1,46E+06
A0A0R4J0G0	*Phosphoenolpyruvate carboxykinase (GTP)*	7	4	73418	4,36E+06		1,46E+06
A0A2I3BQL9	*Phosphoenolpyruvate carboxykinase (GTP)*	8	4	66056	4,36E+06		1,46E+06
Q8BH04	*Phosphoenolpyruvate carboxykinase [GTP], mitochondrial*	7	4	70528	4,36E+06		1,46E+06
Q9D0F9	*Phosphoglucomutase-1*	4	1	61418	9,38E+05	6,91E+05	4,64E+05
Q8BZF8	*Phosphoglucomutase-like protein 5*	27	12	62220	2,43E+07	1,72E+07	1,57E+07
P09411	*Phosphoglycerate kinase 1*	41	10	44,55	4,54E+07	3,59E+07	3,21E+07
P09041	*Phosphoglycerate kinase 2*	12	1	44853			6,23E+05
Q9DBJ1	*Phosphoglycerate mutase 1*	47	8	28,832	2,12E+07	2,10E+07	2,93E+07
Q9Z0Y2	*Phospholipase A2*	29	3	16290	5,52E+06	2,12E+07	2,30E+07
A0A0N4SV32	*Plasminogen activator inhibitor 1 RNA-binding protein*	22	7	39645	4,49E+06	1,11E+06	3,18E+06
Q3UMP4	*Plasminogen activator inhibitor 1 RNA-binding protein*	20	7	44005	4,49E+06	1,11E+06	3,18E+06
Q9CY58	*Plasminogen activator inhibitor 1 RNA-binding protein*	20	7	44714	4,49E+06	1,11E+06	3,18E+06
Q3V0K9	*Plastin-1*	13	5	70408	1,49E+06	1,62E+05	
Q61233	*Plastin-2*	7	2	70149	4,97E+05	6,26E+05	9,41E+05

Q99K51	*Plastin-3*	8	1	70742	2,07E+05		1,08E+05
B1AX58	*Plastin-3*	8	1	71746	2,07E+05		1,08E+05
A0A1C7CYV0	*Plastin-3 (Fragment)*	8	1	70643	2,07E+05		1,08E+05
E9Q3W4	*Plectin*	12	46	499110	1,91E+07	1,48E+07	7,57E+06
Q9QXS1	*Plectin*	11	46	534193	1,91E+07	1,48E+07	7,57E+06
P60335	*Poly(rC)-binding protein 1*	13	2	37498	1,12E+06	6,65E+05	3,65E+06
A0A2R8VI25	*Poly(rC)-binding protein 2*	23	4	33299	2,17E+06	3,13E+06	1,99E+06
Q3TT81	*Poly(rC)-binding protein 2*	23	4	33926	2,17E+06	3,13E+06	1,99E+06
A0A2R8VHP9	*Poly(rC)-binding protein 2*	22	4	35347	2,17E+06	3,13E+06	1,99E+06
B2M1R7	*Poly(rC)-binding protein 2*	20	4	38151	2,17E+06	3,13E+06	1,99E+06
Q61990	*Poly(rC)-binding protein 2*	20	4	38222	2,17E+06	3,13E+06	1,99E+06
A3KFU5	*Polyadenylate-binding protein*	17	7	69430	4,96E+06	6,75E+05	5,22E+06
Q6PHQ9	*Polyadenylate-binding protein*	16	7	72242	4,96E+06	6,75E+05	5,22E+06
P17225	*Polypyrimidine tract-binding protein 1*	13	4	59322	5,10E+06	4,31E+06	5,34E+06
P0CG49	*Polyubiquitin-B*	16	5	34369	1,11E+08	1,12E+08	1,27E+08
Q5SX22	*Polyubiquitin-B (Fragment)*	21	5	26620	1,11E+08	1,12E+08	1,27E+08
P0CG50	*Polyubiquitin-C*	7	5	82550	1,11E+08	1,12E+08	1,27E+08
E9Q5F6	*Polyubiquitin-C (Fragment)*	24	5	22592	1,11E+08	1,12E+08	1,27E+08
Q64436	*Potassium-transporting ATPase alpha chain 1*	42	34	114,065	2,21E+08	3,58E+08	3,07E+08
Q9Z1W8	*Potassium-transporting ATPase alpha chain 2*	3	1	114726		9,60E+05	
P50992	*Potassium-transporting ATPase subunit beta*	30	7	33,566	8,61E+07	1,25E+08	1,05E+08
Q792Y8	*Predicted gene 10334*	28	1	26,119	3,18E+06		3,42E+06
G3UYJ7	*Predicted gene 20441 (Fragment)*	13	1	28642	4,72E+05	1,16E+06	7,33E+05
A0A0G2JEA5	*Predicted gene 43738*	3	2	95223	1,01E+06	3,83E+05	1,10E+06
Q792Y9	*Predicted gene 5771*	32	2	25,993	1,39E+06		4,97E+06
A0A2R8VHP3	*Predicted pseudogene 5478*	11	1	57920	3,03E+06	6,59E+05	2,58E+06
A0A0A6YW67	*Predicted pseudogene 8797*	64	5	8728	1,11E+08	1,12E+08	1,27E+08

Q61838	*Pregnancy zone protein*	17	13	165852	8,94E+06	1,19E+07	1,03E+07
P48678	*Prelamin-A/C*	45	30	74,238	8,73E+07	7,60E+07	6,16E+07
Q61656	*Probable ATP-dependent RNA helicase DDX5*	14	5	69290	1,56E+06	1,14E+06	1,41E+06
Q8BTS0	*Probable ATP-dependent RNA helicase DDX5*	14	5	69266	1,56E+06	1,14E+06	1,41E+06
P62962	*Profilin-1*	66	8	14957	3,16E+07	2,29E+07	2,08E+07
P67778	*Prohibitin 1*	48	11	29820	1,24E+07	1,01E+07	8,90E+06
O35129	*Prohibitin-2*	21	5	33296	7,79E+06	9,35E+06	5,88E+06
P50580	*Proliferation-associated protein 2G4*	19	7	43699	6,18E+06	3,27E+06	6,38E+06
Q91ZA3	*Propionyl-CoA carboxylase alpha chain mitochondrial*	7	4	79922	1,47E+06	1,28E+06	1,62E+06
Q99MN9	*Propionyl-CoA carboxylase beta chain mitochondrial*	18	7	58409	2,86E+06	3,54E+06	3,13E+06
E9PZ00	*Prosaposin*	8	4	60,672	6,89E+05	2,49E+06	3,75E+06
Q8BFQ1	*Prosaposin*	8	4	61,051	6,89E+05	2,49E+06	3,75E+06
K3W4L3	*Prosaposin*	8	4	61,292	6,89E+05	2,49E+06	3,75E+06
J3QPG5	*Prosaposin*	8	4	61,363	6,89E+05	2,49E+06	3,75E+06
Q61207	*Prosaposin*	8	4	61,422	6,89E+05	2,49E+06	3,75E+06
Q91YR9	*Prostaglandin reductase 1*	22	7	35,56	1,66E+07	3,81E+07	3,76E+07
P57096	*Prostate stem cell antigen*	22	2	13478	4,58E+06	1,28E+07	7,81E+06
Q9Z1R9	*Protease, serine 1 (trypsin 1)*	32	1	26,135	1,30E+06	1,48E+07	3,29E+06
Q792Z0	*Protease, serine 3*	28	1	26,184	3,18E+06		3,42E+06
G3UXY0	*Proteasome activator complex subunit 1*	10	2	26092	1,82E+05	1,40E+04	2,24E+05
G3X9K9	*Proteasome activator complex subunit 1*	10	2	27101	1,82E+05	1,40E+04	2,24E+05
P97371	*Proteasome activator complex subunit 1*	9	2	28673	1,82E+05	1,40E+04	2,24E+05
G3UXZ5	*Proteasome activator complex subunit 1 (Fragment)*	10	2	27411	1,82E+05	1,40E+04	2,24E+05
Q9R1P0	*Proteasome subunit alpha type-4*	10	3	29471	1,48E+06	4,65E+06	2,46E+05
Q9Z2U0	*Proteasome subunit alpha type-7*	8	2	27855	1,26E+06		1,36E+06
O55234	*Proteasome subunit beta type-5*	13	3	28532	1,97E+06	2,37E+06	3,41E+06
Q9QXT0	*Protein canopy homolog 2*	14	2	20767	3,65E+06	1,14E+06	1,14E+06

A0A1W2P729	*Protein canopy homolog 2 (Fragment)*	19	2	15316	3,65E+06	1,14E+06	1,14E+06
A2A813	*Protein deglycase*	28	4	18474	2,51E+06	1,93E+06	4,18E+06
P09103	*Protein disulfide-isomerase*	56	30	57,058	3,07E+08	1,07E+08	1,97E+08
A0A0R4J1Y7	*Protein disulfide-isomerase*	27	7	36084	4,87E+06	5,42E+06	3,24E+06
E9PXX7	*Protein disulfide-isomerase*	26	7	38532	4,87E+06	5,42E+06	3,24E+06
D3Z6P0	*Protein disulfide-isomerase A2*	51	25	58,316	3,39E+08	1,70E+08	3,00E+08
P27773	*Protein disulfide-isomerase A3*	50	25	56,678	9,55E+07	8,82E+07	8,17E+07
P08003	*Protein disulfide-isomerase A4*	20	15	71982	1,72E+07	6,59E+06	8,23E+06
A0A0R4J0Z1	*Protein disulfide-isomerase A4*	20	15	72370	1,72E+07	6,59E+06	8,23E+06
Q922R8	*Protein disulfide-isomerase A6*	28	9	48100	4,84E+07	1,36E+07	2,77E+07
Q9D0F3	*Protein ERGIC-53*	13	6	57789	4,22E+07	5,57E+06	5,32E+06
Q9WVE8	*Protein kinase C and casein kinase substrate in neurons protein 2*	13	6	55833	1,87E+06	5,87E+06	1,46E+06
Q3UW53	*Protein Niban 1*	6	4	102649	1,02E+06	1,65E+05	
A6H644	*Protein phosphatase 1 regulatory subunit*	6	3	110972	2,12E+06	7,73E+05	9,70E+05
Q9DBR7	*Protein phosphatase 1 regulatory subunit 12A*	7	6	114996	3,18E+06	8,96E+05	2,12E+06
Q8BG95	*Protein phosphatase 1 regulatory subunit 12B*	6	3	109049	2,12E+06	7,73E+05	9,70E+05
Q9D2Q8	*Protein S100-A14*	21	2	11599	2,31E+06		2,88E+06
P97816	*Protein S100-G*	44	4	8970	8,27E+07	2,28E+06	
Q9EQU5	*Protein SET*	12	3	33378	4,44E+06	1,97E+06	3,15E+06
A2BE92	*Protein SET (Fragment)*	23	3	17584	4,44E+06	1,97E+06	3,15E+06
A2BE93	*Protein SET (Fragment)*	16	3	24924	4,44E+06	1,97E+06	3,15E+06
E9Q1S3	*Protein transport protein SEC23*	5	1	82956	5,71E+05	2,39E+05	#DIV/0!
Q01405	*Protein transport protein Sec23A*	5	1	86162	5,71E+05	2,39E+05	#DIV/0!
Q9D662	*Protein transport protein Sec23B*	9	4	86,437	2,29E+06	2,48E+05	
Q3UPL0	*Protein transport protein Sec31A*	5	5	133569	3,19E+06	4,49E+05	1,03E+06
S4R2A9	*Protein transport protein Sec31A (Fragment)*	7	5	101105	3,19E+06	4,49E+05	1,03E+06
P61620	*Protein transport protein Sec61 subunit alpha isoform 1*	13	6	52265	1,57E+07	5,69E+06	1,05E+07

Q9CQS8	*Protein transport protein Sec61 subunit beta*	26	2	9958	1,05E+07	4,62E+06	9,87E+06
P21981	*Protein-glutamine gamma-glutamyltransferase 2*	28	15	77061	2,22E+07	1,15E+07	1,16E+07
Q08189	*Protein-glutamine gamma-glutamyltransferase E*	8	6	77309	7,31E+06		8,31E+06
P26350	*Prothym*	35	5	12254	9,18E+06	9,34E+06	1,15E+07
Q11011	*Puromycin-sensitive aminopeptidase*	4	3	103325	5,10E+05	1,09E+05	1,76E+05
Q8BK48	*Pyrethroid hydrolase Ces2e*	8	2	62318	2,21E+06		
G5E8R3	*Pyruvate carboxylase*	14	13	129701	5,95E+06	1,21E+07	7,46E+06
E9QPD7	*Pyruvate carboxylase*	14	13	129832	5,95E+06	1,21E+07	7,46E+06
Q05920	*Pyruvate carboxylase mitochondrial*	14	13	129684	5,95E+06	1,21E+07	7,46E+06
P35486	*Pyruvate dehydrogenase E1 component subunit alpha*	16	7	43,232	5,01E+06	9,22E+06	7,34E+06
Q9D051	*Pyruvate dehydrogenase E1 component subunit beta, mitochondrial*	29	8	38,937	6,52E+06	6,03E+06	5,75E+06
E9Q509	*Pyruvate kinase*	6	1	58821	1,35E+06		
G3X925	*Pyruvate kinase*	5	1	62228	1,35E+06		
P53657	*Pyruvate kinase PKLR*	5	1	62309	1,35E+06		
P52480	*Pyruvate kinase PKM*	49	22	57,845	1,20E+08	1,14E+08	1,06E+08
P50396	*Rab GDP dissociation inhibitor alpha*	10	1	50522	2,57E+06	3,18E+05	
Q61598	*Rab GDP dissociation inhibitor beta*	37	9	50537	2,00E+07	1,40E+07	1,07E+07
Q50HX3	*RAB14 protein variant*	25	4	21889	3,72E+06	4,26E+06	2,95E+06
Q5SW88	*RAB1A member RAS oncogene family*	56	5	22372	1,44E+07	7,61E+06	1,07E+07
Q9JKF1	*Ras GTPase-activating-like protein IQGAP1*	8	8	188741	4,71E+06	3,15E+06	1,86E+06
Q3UQ44	*Ras GTPase-activating-like protein IQGAP2*	4	5	180527	6,15E+05	1,53E+06	1,09E+06
Q9D031	*Ras suppressor protein 1*	29	7	31461	3,34E+06	1,60E+06	1,51E+06
Q01730	*Ras suppressor protein 1*	29	7	31550	3,34E+06	1,60E+06	1,51E+06
P61027	*Ras-related protein Rab-10*	26	3	22541	3,05E+06	1,59E+06	1,36E+06
E9Q3P9	*Ras-related protein Rab-11A*	42	7	17375	1,04E+07	1,50E+07	1,23E+07
P62492	*Ras-related protein Rab-11A*	30	7	24394	1,04E+07	1,50E+07	1,23E+07
F8WGS1	*Ras-related protein Rab-11A (Fragment)*	42	7	17668	1,04E+07	1,50E+07	1,23E+07

G3UY29	*Ras-related protein Rab-11B*	44	7	16924	1,04E+07	1,50E+07	1,23E+07
P46638	*Ras-related protein Rab-11B*	30	7	24489	1,04E+07	1,50E+07	1,23E+07
Q91V41	*Ras-related protein Rab-14*	23	4	23897	3,72E+06	4,26E+06	2,95E+06
P62821	*Ras-related protein Rab-1A*	56	5	22678	1,44E+07	7,61E+06	1,07E+07
Q9D1G1	*Ras-related protein Rab-1B*	43	1	22187	4,08E+06	4,61E+06	1,54E+06
A0A494B945	*Ras-related protein Rab-1B (Fragment)*	34	1	28254	4,08E+06	4,61E+06	1,54E+06
P53994	*Ras-related protein Rab-2A*	35	5	23548	4,81E+06	2,08E+06	2,60E+06
P35276	*Ras-related protein Rab-3D*	31	4	24416	4,53E+06	1,28E+06	1,65E+06
P35278	*Ras-related protein Rab-5C*	21	4	23412	2,49E+06	4,58E+06	4,02E+06
Q8C266	*Ras-related protein Rab-5C*	20	4	25352	2,49E+06	4,58E+06	4,02E+06
P51150	*Ras-related protein Rab-7a*	33	6	23490	7,92E+06	9,21E+06	6,89E+06
P55258	*Ras-related protein Rab-8A*	28	2	23668		1,66E+06	8,34E+05
P62835	*Ras-related protein Rap-1A*	25	4	20987	2,98E+06	5,40E+06	6,18E+06
Q99JI6	*Ras-related protein Rap-1b*	25	4	20825	2,98E+06	5,40E+06	6,18E+06
P10833	*Ras-related protein R-Ras*	11	2	23764	1,59E+06	2,05E+06	9,17E+05
G3X8R0	*Receptor expression-enhancing protein*	11	2	21447	6,22E+06	1,65E+06	8,52E+05
A0A494BBE3	*Receptor expression-enhancing protein 5 (Fragment)*	19	2	11535	6,22E+06	1,65E+06	8,52E+05
P97347	*Repetin*	8	7	128619	3,30E+06		1,89E+07
Q99P72	*Reticulon-4*	3	3	126613	9,34E+05	1,15E+06	2,20E+06
Q99PT1	*Rho GDP-dissociation inhibitor 1*	45	8	23,407	7,95E+06	4,12E+06	5,45E+06
Q91VI7	*Ribonuclease inhibitor*	29	10	49817	3,54E+06	4,01E+05	3,17E+06
A0A1B0GSG5	*Ribonuclease inhibitor*	27	10	53926	3,54E+06	4,01E+05	3,17E+06
A0A3B2WBL1	*Ribosomal protein*	27	7	24744	1,53E+07	6,10E+06	1,41E+07
Q5XJF6	*Ribosomal protein*	27	7	24831	1,53E+07	6,10E+06	1,41E+07
I7HLV2	*Ribosomal protein 10*	43	7	23,072	1,57E+07	5,01E+06	5,61E+06
Q9D7S7	*Ribosomal protein eL22-like 1*	16	2	14467	1,41E+07		1,42E+06
A0A1B0GQU8	*Ribosomal protein L18*	39	6	18101	4,16E+07	1,83E+07	3,59E+07

A2A547	*Ribosomal protein L19*	16	2	23248	1,68E+07	1,00E+07	1,57E+07
A0A2I3BPG9	*Ribosomal protein L36A, pseudogene 1*	32	5	12455	1,67E+07	6,02E+06	7,29E+06
A0A1B0GRR3	*Ribosomal protein S11*	27	6	15198	1,15E+07	9,02E+06	9,88E+06
A2AVJ7	*Ribosome binding protein 1*	15	22	158395	2,44E+07	1,32E+07	2,42E+07
Q99PL5	*Ribosome binding protein 2*	14	22	172878	2,44E+07	1,32E+07	2,42E+07
Q9CPN9	*RIKEN cDNA 2210010C04 gene*	21	4	26,422	3,19E+08	5,49E+08	2,86E+08
Q8BTU6	*RNA helicase*	24	1	41290	1,50E+05		
A0A087WPL5	*RNA helicase*	5	7	149618	1,33E+06	1,13E+06	2,75E+06
E9QNN1	*RNA helicase*	5	7	149689	1,33E+06	1,13E+06	2,75E+06
A0A0N4SVP8	*RNA helicase*	10	2	46959		3,36E+05	2,66E+05
Q8CJ40	*Rootletin*	1	2	226942	6,03E+05		
D3Z7V3	*Sarcolemmal membrane-associated protein*	6	4	90721	8,71E+06	8,87E+06	7,06E+06
H7BX64	*Sarcolemmal membrane-associated protein*	5	4	95090	8,71E+06	8,87E+06	7,06E+06
Q3URD3	*Sarcolemmal membrane-associated protein*	5	4	96933	8,71E+06	8,87E+06	7,06E+06
F8WIH0	*Sarcolemmal membrane-associated protein (Fragment)*	7	4	79447	8,71E+06	8,87E+06	7,06E+06
Q8R429	*Sarcoplasmic/endoplasmic reticulum calcium ATPase 1*	10	4	109425	2,29E+06	3,09E+06	3,84E+06
O55143	*Sarcoplasmic/endoplasmic reticulum calcium ATPase 2*	13	7	114,858	5,74E+06	6,71E+06	7,59E+06
Q64518	*Sarcoplasmic/endoplasmic reticulum calcium ATPase 3*	6	1	109529	5,58E+05		
Q6NXL1	*Sec24-related gene family member D (S. cerevisiae)*	2	2	112676	2,64E+06	4,93E+05	1,21E+06
Q63836	*Selenium-binding protein 2*	8	4	52610	2,17E+06	3,71E+06	3,08E+06
E9Q1G8	*Septin*	17	5	50649	1,22E+06	1,10E+06	1,21E+06
P42208	*Septin-2*	14	5	41526	1,23E+06	1,41E+06	1,05E+05
E9Q3V6	*Septin-2*	16	5	36978	1,23E+06	1,41E+06	1,05E+05
D3Z1S1	*Septin-2 (Fragment)*	29	5	20016	1,23E+06	1,41E+06	1,05E+05
F6WYM0	*Septin-2 (Fragment)*	25	5	24258	1,23E+06	1,41E+06	1,05E+05
D3YYB1	*Septin-2 (Fragment)*	24	5	24371	1,23E+06	1,41E+06	1,05E+05
D3Z3C0	*Septin-2 (Fragment)*	24	5	25230	1,23E+06	1,41E+06	1,05E+05

F6UKN5	*Septin-2 (Fragment)*	49	5	12150	1,23E+06	1,41E+06	1,05E+05
F8WIV2	*Serine (or cysteine) peptidase inhibitor clade B member 6a*	9	4	44774	2,53E+06	8,85E+05	5,31E+05
P07759	*Serine protease inhibitor A3K*	44	5	46880	1,17E+07	1,91E+07	1,68E+07
Q6PDM2	*Serine/arginine-rich splicing factor 1*	15	4	27745	2,91E+06	4,02E+06	1,78E+06
H7BX95	*Serine/arginine-rich splicing factor 1*	15	4	28329	2,91E+06	4,02E+06	1,78E+06
P84104	*Serine/arginine-rich splicing factor 3*	19	2	19330	1,61E+06	2,92E+06	2,57E+06
Q8BL97	*Serine/arginine-rich splicing factor 7*	13	2	30818	5,21E+05	5,05E+05	6,92E+05
A0A3Q4EH04	*Serine/arginine-rich-splicing factor 7*	26	2	15763	5,21E+05	5,05E+05	6,92E+05
A0A3Q4L335	*Serine/arginine-rich-splicing factor 7*	16	2	24827	5,21E+05	5,05E+05	6,92E+05
A0A3Q4L393	*Serine/arginine-rich-splicing factor 7*	15	2	26939	5,21E+05	5,05E+05	6,92E+05
P62137	*Serine/threonine-protein ph*	20	1	37540		8,48E+04	
P63330	*Serine/threonine-protein ph*	9	3	35608	2,86E+06	4,01E+06	2,31E+06
P62715	*Serine/threonine-protein ph*	9	3	35575	2,86E+06	4,01E+06	2,31E+06
Q8C483	*Serine--tRNA ligase*	13	6	61167	4,22E+06	9,80E+05	2,53E+06
P26638	*Serine--tRNA ligase cytoplasmic*	13	6	58389	4,22E+06	9,80E+05	2,53E+06
Q921I1	*Serotransferrin*	52	22	76724	3,79E+07	3,91E+07	4,42E+07
Q9D7P9	*Serpin B12*	15	6	47,835	9,69E+06	#DIV/0!	7,20E+06
P70124	*Serpin B5*	23	7	42111	1,10E+06		1,96E+06
Q60854	*Serpin B6*	10	4	42599	2,53E+06	8,85E+05	5,31E+05
P19324	*Serpin H1*	19	7	46534	1,98E+07	1,15E+07	1,11E+07
Q9JK88	*Serpin I2*	24	10	45776	6,22E+07		2,72E+07
H3BLJ9	*S-formylglutathione hydrolase*	35	6	28554	5,34E+06	7,29E+06	7,23E+06
H3BJL6	*S-formylglutathione hydrolase*	34	6	29347	5,34E+06	7,29E+06	7,23E+06
Q9R0P3	*S-formylglutathione hydrolase*	32	6	31320	5,34E+06	7,29E+06	7,23E+06
H3BKH6	*S-formylglutathione hydrolase*	31	6	32829	5,34E+06	7,29E+06	7,23E+06
H3BL99	*S-formylglutathione hydrolase (Fragment)*	35	1	11888	#DIV/0!		
Q9DBL1	*Short/branched chain specific acyl-CoA dehydrogenase mitochondrial*	19	7	47874	6,01E+06	9,83E+06	7,41E+06

E9Q5L3	*Short/branched chain-specific acyl-CoA dehydrogenase mitochondrial*	17	7	51853	6,01E+06	9,83E+06	7,41E+06
Q07417	*Short-chain specific acyl-CoA dehydrogenase mitochondrial*	12	4	44890	5,44E+05	1,45E+06	8,30E+05
Q3U4F0	*Sideroflexin-3*	10	3	30952	1,82E+06	2,33E+06	1,31E+06
Q91V61	*Sideroflexin-3*	9	3	35406	1,82E+06	2,33E+06	1,31E+06
A0A494BB84	*Sideroflexin-3 (Fragment)*	26	3	12494	1,82E+06	2,33E+06	1,31E+06
P47758	*Signal recognition particle receptor subunit beta*	14	3	29579	2,00E+06		2,22E+06
A2AAN2	*Signal recognition particle subunit SRP68*	6	3	65953	1,94E+06		2,59E+05
Q8BMA6	*Signal recognition particle subunit SRP68*	6	3	70574	1,94E+06		2,59E+05
F8VQC1	*Signal recognition particle subunit SRP72*	6	3	74657	8,22E+05		
O09116	*Small proline-rich protein 3*	46	8	25,241	2,03E+07	2,33E+06	5,93E+07
P97351	*Small ribosomal subunit protein eS1*	37	11	29,885	3,75E+07	1,47E+07	3,31E+07
P63325	*Small ribosomal subunit protein eS10*	39	7	18916	1,84E+07	1,05E+07	1,97E+07
P63323	*Small ribosomal subunit protein eS12*	29	5	14515	9,60E+06	4,25E+06	7,95E+06
P63276	*Small ribosomal subunit protein eS17*	47	4	15,524	8,88E+06	4,90E+05	3,47E+06
Q9CZX8	*Small ribosomal subunit protein eS19*	39	8	16085	3,23E+07	1,92E+07	3,04E+07
Q9CQR2	*Small ribosomal subunit protein eS21*	40	3	9,141	3,70E+06	1,87E+06	3,09E+06
P62849	*Small ribosomal subunit protein eS24*	20	2	15423	1,34E+06	1,01E+06	1,63E+06
P62852	*Small ribosomal subunit protein eS25*	34	6	13742	3,94E+07	2,36E+07	2,39E+07
P62855	*Small ribosomal subunit protein eS26*	40	4	13015	2,68E+07	1,60E+07	2,04E+07
Q6ZWY3	*Small ribosomal subunit protein eS27-like*	23	1	9477	7,82E+06		3,27E+06
G3UYV7	*Small ribosomal subunit protein eS28*	57	3	6,344	2,19E+07	1,41E+07	2,28E+07
P62858	*Small ribosomal subunit protein eS28*	46	3	7,841	2,19E+07	1,41E+07	2,28E+07
P62702	*Small ribosomal subunit protein eS4*	44	14	29598	3,40E+07	1,84E+07	2,82E+07
P62754	*Small ribosomal subunit protein eS6*	24	6	28,681	2,62E+07	1,94E+07	2,08E+07
P62082	*Small ribosomal subunit protein eS7*	39	8	22127	3,66E+07	1,66E+07	2,45E+07
P62242	*Small ribosomal subunit protein eS8*	38	7	24205	3,80E+07	2,05E+07	3,03E+07
P68040	*Small ribosomal subunit protein RACK1*	38	10	35,077	2,61E+07	1,48E+07	1,94E+07

P60867	*Small ribosomal subunit protein uS10*	19	2	13373	4,79E+07	6,50E+06	1,27E+07
P62264	*Small ribosomal subunit protein uS11*	23	3	16273	3,55E+07	1,89E+07	3,19E+07
P62267	*Small ribosomal subunit protein uS12*	29	5	15808	8,43E+06	5,68E+06	7,32E+06
P62270	*Small ribosomal subunit protein uS13*	39	8	17719	4,90E+07	2,50E+07	3,35E+07
F6YVP7	*Small ribosomal subunit protein uS13*	39	8	17672	4,90E+07	2,50E+07	3,35E+07
A0A1Y7VKY1	*Small ribosomal subunit protein uS13*	39	8	17749	4,90E+07	2,50E+07	3,35E+07
Q921R2	*Small ribosomal subunit protein uS15*	46	7	16142	2,60E+07	1,14E+07	2,47E+07
P62301	*Small ribosomal subunit protein uS15*	43	7	17222	2,60E+07	1,14E+07	2,47E+07
P62281	*Small ribosomal subunit protein uS17*	22	6	18431	1,15E+07	9,02E+06	9,88E+06
P62908	*Small ribosomal subunit protein uS3*	64	15	26,674	7,62E+07	3,22E+07	4,95E+07
Q6ZWN5	*Small ribosomal subunit protein uS4*	51	14	22591	5,13E+07	2,92E+07	5,17E+07
P25444	*Small ribosomal subunit protein uS5*	37	11	31231	4,71E+07	2,26E+07	3,65E+07
Q91V55	*Small ribosomal subunit protein uS7*	30	6	22876	1,39E+07	6,65E+06	1,37E+07
P62245	*Small ribosomal subunit protein uS8*	35	5	14840	1,29E+07	6,26E+06	9,74E+06
P14131	*Small ribosomal subunit protein uS9*	52	8	16445	5,24E+07	2,07E+07	5,13E+07
Q921U8	*Smoothelin*	12	9	100289	1,15E+07	7,57E+06	6,35E+06
D3Z3Q3	*Smoothelin*	12	9	103040	1,15E+07	7,57E+06	6,35E+06
A0A0G2JGX4	*Sodium/potassium-transporting ATPase subunit alpha*	9	2	112988			1,49E+06
Q8VCE0	*Sodium/potassium-transporting ATPase subunit alpha*	9	2	115969			1,49E+06
Q8VDN2	*Sodium/potassium-transporting ATPase subunit alpha-1*	20	12	112,982	1,58E+07	1,71E+07	1,21E+07
Q6PIC6	*Sodium/potassium-transporting ATPase subunit alpha-3*	9	2	111691			1,49E+06
P14094	*Sodium/potassium-transporting ATPase subunit beta-1*	16	4	35195	4,57E+06	2,61E+06	1,83E+06
P55012	*Solute carrier family 12 member 2*	2	1	131033	8,70E+05	5,90E+05	3,30E+05
E9QM38	*Solute carrier family 12 member 2*	2	1	130668	8,70E+05	5,90E+05	3,30E+05
Q8VEM8	*Solute carrier family 25 member 3*	35	11	39,632	6,26E+07	6,83E+07	5,76E+07
E9QNA7	*Sorbin and SH3 domain-containing protein 1*	22	14	82875	8,81E+06	4,48E+06	4,62E+06
A0A0R4J1W0	*Sorbin and SH3 domain-containing protein 2*	12	6	69576	7,78E+06	3,20E+06	4,23E+06

B2RXQ9	*Sorbin and SH3 domain-containing protein 2*	11	6	72404	7,78E+06	3,20E+06	4,23E+06
Z4YJR7	*Sorbin and SH3 domain-containing protein 2*	7	6	121432	7,78E+06	3,20E+06	4,23E+06
Q3UTJ2	*Sorbin and SH3 domain-containing protein 2*	6	6	132349	7,78E+06	3,20E+06	4,23E+06
B7ZWM6	*Sorbin and SH3 domain-containing protein 2*	6	6	134243	7,78E+06	3,20E+06	4,23E+06
B9EKP8	*Sorbin and SH3 domain-containing protein 2*	6	6	136197	7,78E+06	3,20E+06	4,23E+06
A0A0R4J1T1	*Sorbin and SH3 domain-containing protein 2 (Fragment)*	6	6	127731	7,78E+06	3,20E+06	4,23E+06
Q64442	*Sorbitol dehydrogenase*	10	3	38249	1,25E+07	9,97E+05	2,07E+06
Q6P069	*Sorcin*	15	3	21627	1,65E+06	2,45E+06	1,20E+06
Q8BGH2	*Sorting and assembly machinery component 50 homolog*	16	6	51864	1,96E+06	1,96E+06	1,27E+06
A3KGU7	*Spectrin alpha chain non-erythrocytic 1*	13	27	285151	1,07E+07	1,06E+07	9,02E+06
A3KGU9	*Spectrin alpha chain non-erythrocytic 1*	13	27	287602	1,07E+07	1,06E+07	9,02E+06
A3KGU5	*Spectrin alpha chain non-erythrocytic 1*	13	27	282893	1,07E+07	1,06E+07	9,02E+06
P16546	*Spectrin alpha chain non-erythrocytic 1*	13	27	284596	1,07E+07	1,06E+07	9,02E+06
E9Q447	*Spectrin alpha chain non-erythrocytic 1*	13	27	285344	1,07E+07	1,06E+07	9,02E+06
Q62261	*Spectrin beta chain non-erythrocytic 1*	9	17	274221	3,43E+06	6,24E+06	3,70E+06
Q9WTX5	*S-phase kinase-associated protein 1*	15	2	18672	1,70E+06		1,80E+06
Q9Z1N5	*Splice*	6	3	49035	2,64E+06	4,25E+05	2,22E+06
Q8VIJ6	*Splicing factor proline- and glutamine-rich*	8	5	75442	3,41E+06	4,58E+06	5,54E+06
Q60598	*Src substrate cortactin*	10	6	61250	1,38E+06	4,92E+06	2,64E+06
Q78PY7	*Staphylococcal nuclease domain-containing protein 1*	31	24	102,088	3,84E+07	9,89E+06	2,34E+07
P38647	*Stress-70 protein mitochondrial*	26	16	73461	3,07E+07	3,03E+07	2,65E+07
Q60864	*Stress-induced-ph*	5	3	62582	6,67E+05	4,69E+05	3,17E+05
Q8K2B3	*Succinate dehydrogenase [ubiquinone] flavoprotein subunit*	22	10	72,585	1,11E+07	2,13E+07	1,59E+07
Q9CQA3	*Succinate dehydrogenase [ubiquinone] iron-sulfur subunit*	17	5	31814	1,09E+07	1,01E+07	6,72E+06
Q9WUM5	*Succinate--CoA ligase [ADP/GDP-forming] subunit alpha*	20	5	36155	5,26E+06	3,06E+06	4,80E+06
Q9Z2I9	*Succinate--CoA ligase [ADP-forming] subunit beta*	20	8	50114	5,84E+06	3,72E+06	2,55E+06
Q9Z2I8	*Succinate--CoA ligase [GDP-forming] subunit beta*	18	7	46840	3,99E+06	3,43E+06	3,78E+06

Q9D0K2	*Succinyl-CoA:3-ketoacid coenzyme A transferase 1*	28	10	55989	1,61E+07	2,77E+07	2,37E+07
Q9R112	*Sulfide:quinone oxidoreductase mitochondrial*	24	8	50282	1,34E+06	3,17E+06	2,03E+06
F6ZKZ3	*Sulfide:quinone oxidoreductase mitochondrial*	36	8	33169	1,34E+06	3,17E+06	2,03E+06
Q3UZZ6	*Sulfotransferase 1 family member D1*	16	4	35083	2,70E+06	1,18E+06	2,46E+06
Q9D939	*Sulfotransferase 1C2*	39	10	34,953	1,35E+07	2,61E+07	2,00E+07
P08228	*Superoxide dismutase [Cu-Zn]*	32	4	15943	3,32E+06	9,39E+06	1,02E+07
P09671	*Superoxide dismutase [Mn] mitochondrial*	17	4	24603	4,01E+06	2,71E+06	4,95E+06
E9QPB2	*Suprabasin*	20	10	70493	1,20E+07		1,07E+07
Q8CIT9	*Suprabasin*	20	10	72334	1,20E+07		1,07E+07
Q64310	*Surfeit locus protein 4*	12	2	30,381	3,49E+05	7,31E+05	
E0CXD9	*Surfeit locus protein 4*	38	2	9,561	3,49E+05	7,31E+05	
Q62465	*Synaptic vesicle membrane protein VAT-1 homolog*	8	3	43097	1,27E+06	9,36E+05	9,66E+05
E9Q1U2	*Synaptopodin-2*	12	10	135757	4,67E+06	1,38E+06	2,11E+06
D3YVV9	*Synaptopodin-2*	12	10	129630	4,67E+06	1,38E+06	2,11E+06
Q8VCK7	*Syncollin*	19	3	14595	1,20E+07		5,89E+06
Q70IV5	*Synemin*	22	31	173208	2,06E+07	1,56E+07	7,56E+06
P26039	*Talin-1*	22	39	269819	5,43E+07	2,08E+07	1,98E+07
A0A087WRZ5	*TAR DNA-binding protein 43*	11	3	32178	1,15E+06	9,14E+05	8,69E+05
Q8R0B4	*TAR DNA-binding protein 43*	11	3	33328	1,15E+06	9,14E+05	8,69E+05
Q8BLD4	*TAR DNA-binding protein 43*	11	3	33596	1,15E+06	9,14E+05	8,69E+05
Q6VYI5	*TAR DNA-binding protein 43*	11	3	34197	1,15E+06	9,14E+05	8,69E+05
Q6VYI4	*TAR DNA-binding protein 43*	11	3	34157	1,15E+06	9,14E+05	8,69E+05
Q921F2	*TAR DNA-binding protein 43*	8	3	44548	1,15E+06	9,14E+05	8,69E+05
A0A087WQA5	*TAR DNA-binding protein 43 (Fragment)*	16	3	22822	1,15E+06	9,14E+05	8,69E+05
P11983	*T-complex protein 1 subunit alpha*	7	4	60449	1,77E+06	1,71E+06	1,33E+06
P80314	*T-complex protein 1 subunit beta*	15	5	57477	1,41E+06	8,73E+05	1,61E+06
G5E839	*T-complex protein 1 subunit delta*	12	5	54862	3,81E+06	3,33E+06	3,25E+06

P80315	*T-complex protein 1 subunit delta*	12	5	58066	3,81E+06	3,33E+06	3,25E+06
P80316	*T-complex protein 1 subunit epsilon*	6	3	59624	1,59E+06	8,87E+05	9,06E+05
A0A0N4SV00	*T-complex protein 1 subunit eta*	10	5	55058	2,07E+06	1,60E+06	1,27E+06
P80313	*T-complex protein 1 subunit eta*	9	5	59652	2,07E+06	1,60E+06	1,27E+06
P80318	*T-complex protein 1 subunit gamma*	11	6	60630	4,11E+06	2,44E+06	3,72E+06
P42932	*T-complex protein 1 subunit theta*	14	7	59556	5,47E+06	4,95E+06	4,27E+06
P80317	*T-complex protein 1 subunit zeta*	16	8	58004	5,90E+06	3,69E+06	3,32E+06
Q80YX1	*Tenascin*	5	10	231805	6,23E+06	1,04E+06	9,62E+06
A0A6I8MWZ2	*Tensin 1*	5	1	197192			1,01E+06
Q8R3G9	*Tetraspanin-8*	10	2	25582		7,17E+06	2,89E+06
Q9R0M5	*Thiamin pyroph*	8	1	27068	4,03E+05		
Q8C1A5	*Thimet oligopeptidase*	5	2	78026	5,36E+05		5,76E+05
A0A0R4IZY0	*Thimet oligopeptidase*	5	2	78027	5,36E+05		5,76E+05
P10639	*Thioredoxin*	21	2	11675	5,72E+07	3,53E+07	3,97E+07
Q9CQM5	*Thioredoxin domain-containing protein 17*	15	2	14015	9,82E+05	6,97E+05	1,26E+06
Q91W90	*Thioredoxin domain-containing protein 5*	21	7	46416	4,87E+06	5,42E+06	3,24E+06
P20108	*Thioredoxin-dependent peroxide reductase mitochondrial*	15	4	28127	3,81E+06	2,55E+06	2,43E+06
P52196	*Thiosulfate sulfurtransferase*	15	3	33466	6,36E+05	1,86E+06	7,41E+05
Q9D0R2	*Threonine--tRNA ligase 1 cytoplasmic*	8	4	83356	4,10E+06	1,77E+05	5,14E+05
Q93092	*Transaldolase*	20	7	37387	1,24E+07	9,21E+06	1,01E+07
A0A1B0GR11	*Transaldolase*	18	7	42151	1,24E+07	9,21E+06	1,01E+07
P42669	*Transcriptional activator protein Pur-alpha*	6	2	34884	8,32E+05	1,39E+06	1,22E+06
E9Q1D5	*Transforming growth factor beta-1-induced transcript 1 protein*	7	2	46006	1,32E+06	9,39E+05	8,03E+05
Q62219	*Transforming growth factor beta-1-induced transcript 1 protein*	6	2	50101	1,32E+06	9,39E+05	8,03E+05
P82198	*Transforming growth factor-beta-induced protein ig-h3*	17	9	74597	8,45E+06	1,81E+06	3,06E+06
P37804	*Transgelin*	82	17	22,576	7,23E+08	5,97E+08	5,66E+08
Q9WVA4	*Transgelin-2*	55	8	22,395	4,36E+07	5,67E+07	4,17E+07

Q01853	*Transitional endoplasmic reticulum ATPase*	35	21	89,322	3,52E+07	2,35E+07	2,24E+07
P40142	*Transketolase*	37	19	67,63	4,11E+07	5,90E+07	4,40E+07
P63028	*Translationally-controlled tumor protein*	21	4	19462	1,13E+07	5,73E+06	1,63E+07
Q9CY50	*Translocon-associated protein subunit alpha*	12	3	32065	1,58E+07	2,07E+06	8,68E+06
A0A286YCT4	*Translocon-associated protein subunit alpha*	11	3	33678	1,58E+07	2,07E+06	8,68E+06
A0A286YCG8	*Translocon-associated protein subunit alpha*	11	3	35558	1,58E+07	2,07E+06	8,68E+06
A0A286YDB7	*Translocon-associated protein subunit alpha (Fragment)*	30	3	13111	1,58E+07	2,07E+06	8,68E+06
Q62186	*Translocon-associated protein subunit delta*	31	4	18937	1,36E+07	2,02E+06	7,47E+06
Q9D8L3	*Translocon-associated protein subunit delta*	31	4	19008	1,36E+07	2,02E+06	7,47E+06
Q08423	*Trefoil factor 1*	29	2	9670	5,29E+07	1,67E+08	1,36E+08
Q8BMS1	*Trifunctional enzyme subunit alpha, mitochondrial*	36	23	82,67	2,87E+07	2,48E+07	3,13E+07
Q99JY0	*Trifunctional enzyme subunit beta, mitochondrial*	32	13	51,386	2,78E+07	2,31E+07	2,98E+07
P17751	*Triosephosphate isomerase*	64	13	26713	5,24E+07	5,42E+07	4,49E+07
A2AIM4	*Tropomy*	56	12	32994	1,47E+08	9,27E+07	1,04E+08
D3Z6I8	*Tropomy*	45	1	28723	3,64E+06	7,59E+06	5,48E+06
E9Q7Q3	*Tropomy*	45	1	28734	3,64E+06	7,59E+06	5,48E+06
Q6IRU2	*Tropomy*	36	7	28468	5,10E+06	6,96E+06	4,74E+06
D3Z2H9	*Tropomyosin 3, related sequence 7*	44	3	28,992	2,79E+06	1,25E+06	1,96E+06
P20801	*Troponin C skeletal muscle*	19	2	18110	7,69E+06	#DIV/0!	
Q792Z1	*Trypsin 10*	21	1	26,221	3,80E+06		2,33E+06
P32921	*Tryptophan--tRNA ligase cytoplasmic*	19	7	54358	2,37E+06	1,07E+06	3,43E+06
A0A0A0MQA5	*Tubulin alpha chain*	37	4	52,905	6,12E+06	5,19E+06	8,54E+06
P68369	*Tubulin alpha-1A chain*	43	2	50,136	1,20E+07	1,08E+07	1,01E+07
P68368	*Tubulin alpha-4A chain*	40	4	49,924	6,12E+06	5,19E+06	8,54E+06
Q7TMM9	*Tubulin beta-2A chain*	41	1	49,907	3,82E+05		2,73E+05
Q9CWF2	*Tubulin beta-2B chain*	41	1	49,953	3,82E+05		2,73E+05
P68372	*Tubulin beta-4B chain*	59	3	49,831	9,83E+06	8,59E+06	1,33E+07

P99024	*Tubulin beta-5 chain*	46	2	49,671	1,47E+07	1,34E+07	1,15E+07
Q9CRB6	*Tubulin polymerization-promoting protein family member 3*	24	4	18965	2,16E+06	6,56E+05	2,44E+04
H3BJ97	*Tubulointerstitial nephritis antigen-like*	7	3	49277	1,11E+06	2,82E+05	6,00E+05
Q99JR5	*Tubulointerstitial nephritis antigen-like*	7	3	52665	1,11E+06	2,82E+05	6,00E+05
D3Z637	*Tumor protein D52*	31	4	17603	1,55E+06	3,01E+06	3,33E+06
E9PUA7	*Tumor protein D52*	26	4	21134	1,55E+06	3,01E+06	3,33E+06
Q62393	*Tumor protein D52*	22	4	24313	1,55E+06	3,01E+06	3,33E+06
F8WHQ1	*Tumor protein D52*	20	4	26927	1,55E+06	3,01E+06	3,33E+06
D3Z125	*Tumor protein D52 (Fragment)*	28	4	19417	1,55E+06	3,01E+06	3,33E+06
D3Z7X7	*Tumor protein D52 (Fragment)*	24	4	22562	1,55E+06	3,01E+06	3,33E+06
Q3U4W8	*Ubiquitin carboxyl-terminal hydrolase*	3	2	93355	9,21E+05	6,27E+05	5,29E+05
P56399	*Ubiquitin carboxyl-terminal hydrolase 5*	3	2	95833	9,21E+05	6,27E+05	5,29E+05
P62983	*Ubiquitin-40S rib*	31	5	17951	1,11E+08	1,12E+08	1,27E+08
E9Q9J0	*Ubiquitin-60S rib*	51	5	10914	1,11E+08	1,12E+08	1,27E+08
P62984	*Ubiquitin-60S rib*	38	5	14728	1,11E+08	1,12E+08	1,27E+08
P68037	*Ubiquitin-conjugating enzyme E2 L3*	40	5	17862	6,12E+06	5,85E+06	5,38E+06
P61089	*Ubiquitin-conjugating enzyme E2 N*	29	4	17138	9,04E+06	7,22E+06	6,52E+06
P61961	*Ubiquitin-fold modifier 1*	51	2	9118	5,19E+06		3,06E+06
Q02053	*Ubiquitin-like modifier-activating enzyme 1*	12	11	117809	1,32E+07	1,02E+07	1,31E+07
O70475	*UDP-gluc*	29	13	54832	7,45E+06	1,51E+07	1,05E+07
Q6P5E4	*UDP-glucose:glycoprotein glucosyltransferase 1*	3	5	176433	2,46E+06		2,74E+07
Q9DBP5	*UMP-CMP kinase*	29	5	22165	7,44E+06	7,35E+05	3,31E+06
A0A0R4J093	*UMP-CMP kinase*	25	5	25715	7,44E+06	7,35E+05	3,31E+06
Q9WTI7	*Unconventional my*	12	10	121944	2,19E+06	2,45E+06	2,87E+06
Q91ZJ5	*UTP-glucose-1-phospate uridylytransferase*	12	4	56979	2,46E+06	1,62E+06	1,36E+06
Q9Z1Q9	*Valine--tRNA ligase*	10	10	140215	4,74E+06	4,49E+05	1,74E+06
P50544	*Very long-chain specific acyl-CoA dehydrogenase mitochondrial*	22	11	70876	4,72E+06	3,90E+06	4,87E+06

Q8VDJ3	*Vigilin*	19	21	141742	1,99E+07	4,70E+06	1,27E+07
Q62468	*Villin-1*	9	7	92775	1,72E+07		
P20152	*Vimentin*	50	15	53,688	1,04E+08	8,84E+07	6,39E+07
Q64727	*Vinculin*	42	39	116,717	1,20E+08	8,28E+07	7,37E+07
Q60932	*Voltage-dependent anion-selective channel protein 1*	26	5	32351	2,19E+07	2,43E+07	2,02E+07
G3UX26	*Voltage-dependent anion-selective channel protein 2*	35	8	30446	2,15E+07	2,94E+07	2,40E+07
Q60930	*Voltage-dependent anion-selective channel protein 2*	33	8	31733	2,15E+07	2,94E+07	2,40E+07
Q60931	*Voltage-dependent anion-selective channel protein 3*	12	2	30753	3,45E+06	3,31E+06	1,96E+06
J3QMG3	*Voltage-dependent anion-selective channel protein 3*	12	2	30852	3,45E+06	3,31E+06	1,96E+06
O88342	*WD repeat-containing protein 1*	15	8	66407	7,01E+06	1,09E+07	7,91E+06
Q3UE92	*Xaa-Pro aminopeptidase 1*	10	5	74560	1,68E+06	4,87E+05	4,92E+05
Q6P1B1	*Xaa-Pro aminopeptidase 1*	11	5	69591	1,68E+06	4,87E+05	4,92E+05
P62960	*Y-box-binding protein 1*	9	2	35730	1,16E+06	5,70E+05	7,71E+05
Q8K0C5	*Zymogen granule membrane protein 16*	20	2	18210	1,67E+07		1,31E+07
