# Supplementary material for: Role of Açaí (Euterpe oleracea) in Modulating the Immune Response During Experimental Oral Infection with Trypanosoma cruzi
Source: Microorganisms. 2025 Nov 28;13(12):2711. doi: 10.3390/microorganisms13122711 (PMC12735136; doi:10.3390/microorganisms13122711)
Supplement: Supplementary file 1 [file microorganisms-13-02711-s001.zip › Table S2.pdf]

Table S2. Part I – Proteins uniquely found in Control group

Accession	Description	Coverage (%)	Unique Peptides	Molecular Mass (Da)	Control Area
**P05977**	Myosin l light chain 1/3	35	3	20594	1,95E+07
**E9PWG4**	Myosin l light chain 1/3	40	3	17689	1,95E+07
**Q62468**	Villin-1	9	7	92775	1,72E+07
**F8VQM0**	Alkaline phosphatase	12	4	60291	1,15E+07
**P55050**	Fatty acid-binding protein intestinal	14	2	15126	9,40E+06
**P70695**	Fructose-1 6-bisphosphatase isozyme 2	15	4	36947	8,28E+06
**P20801**	Troponin C skeletal muscle	19	2	18110	7,69E+06
**Q8VCN5**	Cystathionine gamma-lyase	13	5	43567	7,23E+06
**Q99JW5**	Epithelial cell adhesion molecule	6	1	35019	4,45E+06
**Q9D2R4**	Transmembrane emp24 domain-containing protein 11	10	2	24842	4,35E+06
**P47856**	Glutamine--fructe-6-phosphate aminotransferase	4	3	78539	4,23E+06
**Q99PM0**	Microfibril-associated glycoprotein 1	5	1	20781	4,16E+06
**P55002**	Microfibrillar-associated protein 2	5	1	20578	4,16E+06
**Q9CZS1**	Aldehyde dehydrogenase X mitochondrial	10	3	57553	3,89E+06
**Q9R100**	Cadherin-17	3	2	91645	3,45E+06
**A2AKS7**	Cadherin-17	3	2	80655	3,45E+06
**Q9EPC1**	Alpha-parvin	6	2	42330	3,26E+06
**O09165**	Calsequestrin-1	6	2	46378	3,21E+06
**P04247**	Myoglobin	23	2	17070	2,81E+06
**A0A571BF58**	Nebulin	0	1	871252	2,76E+06
**A0A571BF46**	Nebulin	0	1	867113	2,76E+06
**E9Q1W3**	Nebulin	0	1	828679	2,76E+06

**A2AQA9**	Nebulin	0	1	800586	2,76E+06
**E9Q264**	Myosin heavy chain 15	2	2	221844	2,76E+06
**Q8BK48**	Pyrethroid hydrolase Ces2e	8	2	62318	2,21E+06
**O08691**	Arginase-2 mitochondrial	9	2	38878	1,83E+06
**Q62446**	Peptidyl-prolyl cis-trans isomerase FKBP3	5	1	25148	1,67E+06
**Q91V04**	Translocating chain-associated membrane protein 1	3	1	43039	1,60E+06
**Z4YKB8**	Heterochromatin protein 1-binding protein 3	2	1	60851	1,59E+06
**Q62318**	Transcription intermediary factor 1-beta	3	2	88847	1,44E+06
**Q64133**	Amine oxidase [flavin-containing] A	5	2	59602	1,38E+06
**P23492**	Purine nucleoside phphorylase	3	1	32277	1,37E+06
**Q543K9**	Purine nucleoside phphorylase	3	1	32263	1,37E+06
**F8VPQ6**	Alkaline phosphatase	7	1	59532	1,32E+06
**D3YTQ9**	40S ribosomal protein S15	11	1	13742	1,21E+06
**P62843**	40S ribosomal protein S15	9	1	17040	1,21E+06
**Q8JZR0**	Long-chain-fatty-acid--CoA ligase 5	4	1	76206	1,19E+06
**Q3U125**	Peroxiredoxin-like 2A	10	2	25682	1,18E+06
**Q9CYH2**	Peroxiredoxin-like 2A	11	2	24395	1,18E+06
**P48024**	Eukaryotic translation initiation factor 1	12	1	12747	1,18E+06
**J3QPZ8**	Eukaryotic translation initiation factor 1 (Fragment)	13	1	12100	1,18E+06
**A0A1L1SSA3**	Eukaryotic translation initiation factor 1b (Fragment)	14	1	11358	1,18E+06
**J3QN87**	Eukaryotic translation initiation factor 1 (Fragment)	16	1	10364	1,18E+06
**Q9CXU9**	Eukaryotic translation initiation factor 1b	12	1	12824	1,18E+06
**Q8C196**	**Carbamoyl-phosphate** synthase [ammonia] mitochondrial	1	1	164617	1,06E+06
**Q9QUI0**	Transforming protein RhoA	6	1	21782	1,00E+06
**O89094**	Caspase-14	11	2	29458	9,24E+05
**F8VQC1**	Signal recognition particle subunit SRP72	6	3	21	8,22E+05

**P38060**	Hydroxymethylglutaryl-CoA lyase mitochondrial	3	1	34239	8,13E+05
**Q8VDM4**	26S proteasome non-ATPase regulatory subunit 2	3	3	100203	7,99E+05
**E9PYJ9**	LIM domain-binding protein 3	2	1	72312	7,52E+05
**E9Q559**	Calcium-transporting ATPase	6	1	112504	5,58E+05
**B1ATS5**	Calcium-transporting ATPase	6	1	111709	5,58E+05
**Q64518**	Sarcoplasmic/endoplasmic reticulum calcium ATPase 3	6	1	109529	5,58E+05
**B1ATS4**	Calcium-transporting ATPase	6	1	107600	5,58E+05
**A0A0J9YUL3**	Septin	5	1	49782	4,54E+05
**Q8C1B7**	Septin-11	5	1	49695	4,54E+05
**A0A0J9YTY0**	Septin	5	1	48979	4,54E+05
**Q9R0M5**	Thiamin pyrophphokinase 1	8	1	27068	4,03E+05
**E9PZW8**	Unconventional myosin-IXb	0	1	240314	3,70E+05
**Q9QY06**	Unconventional myosin-IXb	0	1	238832	3,70E+05
**E9PWZ6**	Unconventional myosin-IXb	0	1	223924	3,70E+05
**A0A1D5RLD1**	Unconventional myosin-IXb	0	1	222640	3,70E+05
**E9QKV6**	Unconventional myosin-IXb	0	1	222440	3,70E+05
**Q9QZ06**	Toll-interacting protein	5	1	30345	3,65E+05
**Q8C5G6**	Toll-interacting protein	6	1	24540	3,65E+05
**Q9CR60**	Vesicle transport protein GOT1B	10	1	15422	3,38E+05
**A0A1D5RLM8**	Predicted gene 11639	0	1	654216	3,26E+05
**O55135**	Eukaryotic translation initiation factor 6	4	1	26511	3,24E+05
**Q7M6Y3**	Phphatidylinitol-binding clathrin assembly protein	5	1	71543	3,02E+05
**K3W4L7**	Phenazine biosynthesis-like domain-containing protein 1	4	1	32190	2,86E+05
**Q9DCG6**	Phenazine biosynthesis-like domain-containing protein 1	4	1	32048	2,86E+05

**Q9CXN7**	Phenazine biosynthesis-like domain-containing protein 2	4	1	31983	2,86E+05
**A0A1W2P7N2**	Phenazine biosynthesis-like domain-containing protein 1	9	1	14765	2,86E+05
**A0A1W2P711**	Melanoma inhibitory activity protein 2	1	1	156590	2,74E+05
**Q91ZV0**	Melanoma inhibitory activity protein 2	1	1	156461	2,74E+05
**A0A5F8MPH8**	Tenascin XB	0	1	474067	2,62E+05
**A0A571BG59**	Tenascin XB	0	1	447177	2,62E+05
**O35452**	Tenascin X	0	1	435481	2,62E+05
**E9Q2T3**	Tenascin XB	0	1	339905	2,62E+05
**Q9QXC1**	Fetuin-B	2	1	42713	2,50E+05
**Q6YJU1**	Fetuin-B	3	1	33880	2,50E+05
**Q8K010**	5-oxoprolinase	2	2	137611	2,24E+05
**A2A702**	Eukaryotic translation initiation factor 3 subunit M	4	1	27879	1,50E+05
**Q99JX4**	Eukaryotic translation initiation factor 3 subunit M	3	1	42517	1,50E+05
**A2AH25**	Rho GTPase-activating protein 1	2	1	54437	1,38E+05
**Q5FWK3**	Rho GTPase-activating protein 1	2	1	50411	1,38E+05
**P03921**	NADH-ubiquinone oxidoreductase chain 5	3	1	68475	1,33E+05
**P58389**	Serine/threonine-protein phosphatase 2A activator	3	1	36710	1,06E+05
**Q3TXS7**	26S proteasome non-ATPase regulatory subunit 1	1	1	105730	9,86E+04
**Q9JM83**	Calmodulin-4	8	1	16767	1,75E+04
**A2AQP0**	Myin-7B	4	1	221495	
**D3Z5G7**	Carboxylic ester hydrolase	6	1	62197	

Table S2. Part II – Proteins uniquely found in RPMI group

Accession	Description	Coverage (%)	Unique Peptides	Molecular Mass (Da)	RPMI Area
**E9Q0T8**	Dynein axonemal heavy chain 7A	0	1	460908	1,74E+07
**L7N1Y0**	Dynein axonemal heavy chain 7B	0	1	466694	1,74E+07
**A0A571BD48**	Dynein axonemal heavy chain 7B	0	1	461919	1,74E+07
**G3UXL2**	Ribe-phosphate diphosphokinase	4	1	34824	3,08E+06
**E9Q7N9**	Dynein axonemal heavy chain 11	0	1	516323	1,48E+06
**Q9D8I3**	Glyoxalase domain-containing protein 5	9	1	16595	1,22E+06
**Q9D023**	Mitochondrial pyruvate carrier 2	17	2	14286	1,02E+06
**E9Q641**	Nebulin-related-anchoring protein	1	1	167519	8,86E+05
**A0A0U1RPJ3**	Perilipin-1 (Fragment)	5	1	20135	6,15E+05
**Q8CGN5**	Perilipin-1	2	1	55596	6,15E+05
**Q8BI08**	Protein MAL2	6	1	19094	4,65E+05
**P63321**	Ras-related protein Ral-A	10	1	23553	4,17E+05
**A0A1Y7VL93**	Small monomeric GTPase (Fragment)	15	1	15353	4,17E+05
**P58871**	182 kDa tankyrase-1-binding protein	1	1	181824	3,58E+05
**Z4YJL4**	182 kDa tankyrase-1-binding protein	1	1	112648	3,58E+05
**P58871**	182 kDa tankyrase-1-binding protein	1	1	181824	3,58E+05
**Z4YJL4**	182 kDa tankyrase-1-binding protein	1	1	112648	3,58E+05
**A0A3B2WBH9**	Tight junction protein ZO-2	1	1	131106	2,72E+05
**P12815**	Programmed cell death protein 6	5	1	21867	2,69E+05
**P49586**	**Choline-phosphate** cytidylyltransferase A	4	1	41667	2,67E+05
**D3Z3T5**	**Choline-phosphate** cytidylyltransferase	6	1	29761	2,67E+05
**Q9JIG8**	PRA1 family protein 2	6	1	19478	2,67E+05
**B1AQZ0**	Septin-8	4	1	55874	2,16E+05

**B1AQY9**	Septin	4	1	51146	2,16E+05
**B7ZC46**	Septin	4	1	50896	2,16E+05
**Q8CHH9**	Septin-8	4	1	49812	2,16E+05
**Q8R180**	ERO1-like protein alpha	3	1	1	1,97E+05
**O54692**	Centromere/kinetochore protein zw10 homolog	1	1	88063	1,87E+05
**Q9WUQ2**	Prolactin regulatory element-binding protein	2	1	45437	1,84E+05
**D3Z3S1**	Prolactin regulatory element-binding protein	3	1	37965	1,84E+05
**Q91XH5**	Sepiapterin reductase	6	1	27928	9,27E+04
**Q64105**	Sepiapterin reductase	6	1	27883	9,27E+04
**G3UXX3**	Sepiapterin reductase	7	1	23357	9,27E+04
**Q62148**	Retinal dehydrogenase 2	4	1	56626	7,59E+04
**Q9JHW9**	Aldehyde dehydrogenase family 1 member A3	4	1	56157	7,59E+04

Table S2. Part III – Proteins uniquely found in Açaí group.

Accession	Description	Coverage (%)	Unique Peptides	Molecular Mass (Da)	Açaí Area
**Q9QXK3**	Coatomer subunit gamma-2	3	1	97680	6,56E+06
**Q9QX97**	Trefoil Factor 2/Spasmolytic polypeptide protein	12	1	14271	1,98E+06
**Q03404**	Trefoil factor 2	12	1	14172	1,98E+06
**Q5U4B1**	Replication factor C subunit 1	1	1	126064	1,72E+06
**A0A0N5E9G7**	Replication factor C subunit 1	1	1	125865	1,72E+06
**Q9CQN1**	Heat shock protein 75 kDa mitochondrial	3	1	80209	1,24E+06
**Q9EST1**	Gasdermin-A	11	1	49593	1,12E+06
**P61087**	Ubiquitin-conjugating enzyme E2 K	14	2	22407	1,11E+06
**A0A494BB95**	Eukaryotic translation initiation factor 4C	4	1	28613	1,09E+06

**Q60872**	Eukaryotic translation initiation factor 1A	8	1	16502	1,09E+06
**Q8BMJ3**	Eukaryotic translation initiation factor 1A X-chromosomal	8	1	16460	1,09E+06
**Q8R2P8**	Lysine--tRNA ligase	4	1	71291	1,07E+06
**A0A6I8MWZ2**	Tensin 1	5	1	197192	1,01E+06
**Q8C6B0**	Methyltransferase-like 7A1	10	1	28096	8,60E+05
**A0A6I8MWZ0**	Peptidylprolyl isomerase (Fragment)	1	1	114364	6,79E+05
**A2AMT1**	Filensin	1	1	73669	6,67E+05
**Q8BGQ7**	Alanine--tRNA ligase cytoplasmic	1	1	106908	6,54E+05
**P97855**	Ras GTPase-activating protein-binding protein 1	3	1	51829	6,31E+05
**Q5SWT3**	Solute carrier family 25 member 35	4	1	32631	6,15E+05
**A0A0A0MQ70**	Solute carrier family 25 member 35	4	1	32167	6,15E+05
**A0A1B0GRP7**	Pyridoxal phosphate hometasis protein (Fragment)	4	1	36180	4,16E+05
**Q9Z2Y8**	Pyridoxal phosphate hometasis protein	5	1	30049	4,16E+05
**Q6P8V7**	Proline synthetase co-transcribed	8	1	16937	4,16E+05
**A0A1B0GRR5**	Pyridoxal phosphate hometasis protein (Fragment)	9	1	15275	4,16E+05
**A0A1B0GQY5**	Pyridoxal phosphate hometasis protein	10	1	14280	4,16E+05
**A0A1B0GSA1**	Pyridoxal phosphate hometasis protein (Fragment)	19	1	7724	4,16E+05
**Q8K1Z0**	Ubiquinone biosynthesis protein COQ9 mitochondrial	3	1	35083	3,75E+05
**F6SFF5**	Ubiquinone biosynthesis protein (Fragment)	3	1	30282	3,75E+05
**B0R091**	Calcineurin B homologous protein 1	6	1	21998	3,40E+05
**O88668**	Protein CREG1	5	1	24452	2,78E+05
**K4DI63**	Protein CREG1	12	1	11021	2,78E+05
**Q2TPA8**	Hydroxysteroid dehydrogenase-like protein 2	2	1	54208	2,40E+05
**B1AX78**	Hydroxysteroid dehydrogenase-like protein 2	2	1	41866	2,40E+05
**Q6PA06**	Atlastin-2	3	1	66224	2,09E+05
**E9QND8**	Atlastin-2	4	1	46876	2,09E+05
**P62196**	26S proteasome regulatory subunit 8	6	1	45626	2,03E+05

**Q8K1K2**	26S proteasome regulatory subunit 8	7	1	38752	2,03E+05
**Q99KK2**	N-acylneuraminate cytidylyltransferase	3	1	48058	1,53E+05
**A0A0M3HEP8**	Vitamin-K-epoxide reductase (warfarin-sensitive)	8	1	14664	1,49E+05
**Q6TEK5**	Vitamin K epoxide reductase complex subunit 1-like protein 1	6	1	19779	1,49E+05
**Q9DCG9**	Multifunctional methyltransferase subunit TRM112-like protein	10	1	14141	1,22E+05
**A2AAJ9**	Obscurin	0	1	966606	6,00E+04
**H7BX05**	Non-specific serine/threonine protein kinase	0	1	874552	6,00E+04
**E9QQ96**	Non-specific serine/threonine protein kinase	0	1	815649	6,00E+04
**Z4YJE4**	Non-specific serine/threonine protein kinase	0	1	781586	6,00E+04
**A0A1W2P6H1**	Non-specific serine/threonine protein kinase	0	1	725792	6,00E+04
**Q9QYR9**	Acyl-coenzyme A thioesterase 2 mitochondrial	2	1	49657	4,95E+04
**Q6Q2Z6**	Acyl-coenzyme A thioesterase 5	3	1	46573	4,95E+04
