# Supplementary material for: Role of Açaí (Euterpe oleracea) in Modulating the Immune Response During Experimental Oral Infection with Trypanosoma cruzi
Source: Microorganisms. 2025 Nov 28;13(12):2711. doi: 10.3390/microorganisms13122711 (PMC12735136; doi:10.3390/microorganisms13122711)
Supplement: Supplementary file 1 [file microorganisms-13-02711-s001.zip › Table S3.pdf]

Table S3. Differentially expressed proteins identified in quantitative analysis.

Upregulated Proteins in the Açaí group and downregulated in the RPMI group.							
Accesssion	Description	Coverage (%)	Unique Peptides	Control Area	Açaí Area	RPMI Area	Ratio C:A:R
Q9CR35	Chymotrypsinogen B	37	8	9,49E+07	1,08E+08	2,39E+05	1.00:1.13:0.00
P05208	Chymotrypsin-like elastase family member 2A	46	8	2,48E+07	3,40E+07	5,13E+04	1.00:1.37:0.00
P00688	Pancreatic alpha-amylase	48	13	8,08E+07	1,25E+08	6,61E+05	1.00:1.54:0.01
Q9ER05	Chymopasin	30	5	1,41E+07	3,05E+07	6,43E+04	1.00:2.15:0.00
Q9Z2U0	Proteasome subunit alpha type-7	8	2	1,55E+06	2,48E+06	5,79E+05	1.00:1.60:0.37
Q05816	Fatty acid-binding protein 5	48	6	6,25E+07	6,63E+07	2,31E+07	1.00:1.06:0.37
F8VQM0	Alkaline phosphatse	15	4	6,21E+06	1,01E+07	9,79E+05	1.00:1.63:0.16
D3YYE1	Acidic leucine-rich nuclear phosphoprotein	22	3	4,88E+06	6,64E+06	8,96E+05	1.00:1.36:0.18
Q5DTZ0	Protein NYNRIN	1	2	9,95E+05	3,50E+06	5,64E+05	1.00:3.52:0.57
O09116	Small proline-rich protein 3	46	8	1,15E+07	3,57E+07	7,44E+05	1.00:3.10:0.06
Q9CYH2	Peroxiredoxin-like 2A	15	3	1,40E+06	8,11E+06	2,18E+05	1.00:5.79:0.16
P18165	Loricrin	5	2	3,67E+06	1,67E+07	2,03E+04	1.00:4.55:0.01
P97347	Repetin	8	8	4,19E+06	7,34E+06	4,81E+05	1.00:1.75:0.11
Q62266	Cornifin-A	22	4	2,78E+07	5,23E+07	5,06E+06	1.00:1.88:0.18
Q9ERE2	Keratin type II cuticular Hb1	20	2	1,77E+06	2,59E+06	8,73E+04	1.00:1.47:0.05
E9QLW7	Dermokine	10	5	2,67E+06	4,30E+06	1,12E+05	1.00:1.61:0.04
P04104	Keratin, type II cytoskeletal 1	61	31	1,85E+08	3,13E+08	4,00E+07	1.00:1.69:0.22
A0A0A6YVU7	Filaggrin	29	4	5,34E+07	9,57E+07	8,64E+06	1.00:1.79:0.16
Q6IME9	Keratin type II cytoskeletal 72	9	1	1,25E+07	2,38E+07	1,96E+06	1.00:1.90:0.16
A2A513	Keratin 10	47	23	2,35E+08	3,51E+08	4,58E+07	1.00:1.50:0.20
P03958	Adenosine deaminase	52	14	2,15E+07	2,46E+07	1,78E+06	1.00:1.15:0.08
Q9CRB1	Galectin	68	7	5,73E+07	6,71E+07	4,83E+06	1.00:1.17:0.08

Q8VHD8	Hornerin	5	7	7,86E+06	9,91E+06	1,14E+06	1.00:1.26:0.14
Q08879	Fibulin-1	3	2	6,51E+05	9,31E+05	9,47E+04	1.00:1.43:0.15

Downregulated proteins in both infected groups.

Accession	Description	Coverage (%)	Unique Peptides	Control Area	Açaí Area	RPMI Area	Ratio C:A:R
P97816	Protein S100-G	44	4	2,56E+07	8,09E+04	1,75E+06	1.00:0.00:0.07
A8Y5N4	17-beta-hydroxysteroid dehydrogenase 13	19	3	1,46E+06	7,30E+05	2,43E+05	1.00:0.50:0.17
Q61753	D-3-phosphoglycerate dehydrogenase	20	9	8,31E+06	4,20E+06	1,36E+06	1.00:0.51:0.16
Q64285	Bile salt-activated lipase	23	13	2,60E+07	2,16E+07	1,87E+05	1.00:0.83:0.01
P27661	Histone H2AX	28	1	1,87E+06	1,23E+06	4,30E+05	1.00:0.66:0.23
Q7TSV4	Phosphopentomutase	4	2	1,09E+06	3,97E+05	2,14E+05	1.00:0.36:0.20
P62849	Small ribosomal subunit protein eS24	20	2	6,14E+06	5,45E+06	1,13E+06	1.00:0.89:0.18
O70456	14-3-3 protein sigma	51	8	7,69E+06	6,12E+06	1,82E+06	1.00:0.80:0.24
D3YU60	Microsomal glutathione S-transferase 1	13	1	8,60E+05	5,73E+05	5,22E+04	1.00:0.67:0.06
P07744	Keratin type II cytoskeletal 4	53	29	2,03E+08	1,55E+08	1,84E+07	1.00:0.76:0.09
E9Q1Y3	Apolipoprotein B-100 (Fragment)	0	2	1,48E+07	4,51E+06	6,61E+05	1.00:0.30:0.04
P08730	Keratin, type I cytoskeletal 13	54	16	1,75E+08	1,06E+08	1,62E+07	1.00:0.61:0.09
Q9JHR7	Insulin-degrading enzyme	3	3	1,09E+06	5,32E+05	2,99E+04	1.00:0.49:0.03
Q9D7P9	Serpin B12	21	8	7,62E+06	4,47E+06	1,93E+05	1.00:0.59:0.03
Q922U2	Keratin, type II cytoskeletal 5	46	19	3,56E+07	3,07E+07	6,31E+06	1.00:0.86:0.18
Q08189	Protein-glutamine gamma-glutamyltransferase E	12	8	4,99E+06	3,02E+06	4,25E+05	1.00:0.61:0.09
E9Q0F0	Keratin 78	23	22	2,18E+07	1,82E+07	4,06E+06	1.00:0.83:0.19

Upregulated proteins in infected groups.

Accession	Description	Coverage (%)	Unique Peptides	Control Area	Açaí Area	RPMI Area	Ratio C:A:R
P32261	Antithrombin-III	7	3	7,57E+05	2,00E+06	3,36E+06	1.00:2.64:4.44

A2A6H0	LIM and SH3 domain protein 1 (Fragment)	33	7	1,54E+06	4,65E+06	7,93E+06	1.00:3.03:5.17
P57096	Prostate stem cell antigen	22	2	4,92E+06	1,11E+07	1,85E+07	1.00:2.26:3.76
P45481	Histone lysine acetyltransferase CREBBP	1	2	5,87E+05	7,97E+05	3,20E+06	1.00:1.36:5.45
Q9CQW5	Galectin-2	66	8	2,22E+07	3,97E+07	7,99E+07	1.00:1.79:3.60
Q99KE1	NAD-dependent malic enzyme mitochondrial	7	3	5,47E+05	2,17E+06	4,89E+06	1.00:3.96:8.94
P13745	Glutathione S-transferase A1	27	6	6,31E+07	1,00E+08	1,66E+08	1.00:1.59:2.63
Q9CQS6	Gastrokine-2	39	8	8,15E+07	1,75E+08	2,72E+08	1.00:2.15:3.33
E9QAQ8	Mucin 5 subtypes A and C tracheobronchial/gastric	10	29	4,54E+06	1,98E+07	2,07E+07	1.00:4.36:4.56
Q9CR36	Gastrokine-1	40	7	1,41E+08	3,80E+08	4,36E+08	1.00:2.69:3.08
Q08423	Trefoil factor 1	53	3	5,84E+07	1,87E+08	2,44E+08	1.00:3.19:4.18
Q8K354	Carbonyl reductase [NADPH] 3	35	6	1,64E+06	5,54E+06	6,83E+06	1.00:3.37:4.16
Q91XV3	Brain acid soluble protein 1	22	3	4,03E+05	1,66E+06	2,52E+06	1.00:4.11:6.27
Q9D7R7	Gastricsin	7	3	5,90E+07	1,44E+08	1,60E+08	1.00:2.44:2.71
Q9CQC2	Colipase	50	5	3,88E+07	1,26E+08	1,01E+08	1.00:3.24:2.61
Q8VHB5	Carbonic anhydrase 9	7	3	1,96E+05	1,60E+06	1,63E+06	1.00:8.14:8.31
Q91XA9	Acidic mammalian chitinase	26	10	3,78E+07	1,46E+08	1,04E+08	1.00:3.85:2.75

Downregulate proteins in Açaí group and upregulate in RPMI group.

Accession	Description	Coverage (%)	Unique Peptides	Control Area	Açaí Area	RPMI Area	Ratio C:A:R
A0A1W2P756	Mitochondrial import inner membrane translocase subunit Tim13	33	2	2,53E+06	5,12E+05	6,11E+06	1.00:0.20:2.41
P16015	OCarbonic anhydrase 3	32	6	8,47E+06	2,42E+06	1,59E+07	1.00:0.29:1.87
Q3ULJ0	Glycerol-3-phosphate dehydrogenase 1-like protein	8	2	8,96E+05	2,21E+05	5,13E+06	1.00:0.25:5.73
